# Recent advances in transition metal-catalyzed Csp^2^-monofluoro-, difluoro-, perfluoromethylation and trifluoromethylthiolation

**DOI:** 10.3762/bjoc.9.287

**Published:** 2013-11-15

**Authors:** Grégory Landelle, Armen Panossian, Sergiy Pazenok, Jean-Pierre Vors, Frédéric R Leroux

**Affiliations:** 1 CNRS-Université de Strasbourg, UMR 7509, SynCat, ECPM, 25 Rue Becquerel, 67087 Strasbourg Cedex 02, France; 2 Bayer CropScience AG, Alfred-Nobel-Strasse 50, 40789 Monheim, Germany; 3 Bayer SAS, 14 impasse Pierre Baizet, 69263 Lyon, Cedex 09, France

**Keywords:** catalysis, cross-coupling, difluoromethylation, fluorine, monofluoromethylation, organo-fluorine, transition metal, trifluoromethylation, trifluoromethylthiolation

## Abstract

In the last few years, transition metal-mediated reactions have joined the toolbox of chemists working in the field of fluorination for Life-Science oriented research. The successful execution of transition metal-catalyzed carbon–fluorine bond formation has become a landmark achievement in fluorine chemistry. This rapidly growing research field has been the subject of some excellent reviews. Our approach focuses exclusively on transition metal-catalyzed reactions that allow the introduction of –CFH_2_, –CF_2_H, –C*_n_*F_2_*_n_*_+1_ and –SCF_3_ groups onto sp² carbon atoms. Transformations are discussed according to the reaction-type and the metal employed. The review will not extend to conventional non-transition metal methods to these fluorinated groups.

## Review

### Introduction

The incorporation of fluorine or fluorinated moieties into organic compounds plays a key role in Life-Science oriented research as often-profound changes of the physico-chemical and biological properties can be observed [1–6]. As a consequence, organofluorine chemistry has become an integral part of pharmaceutical [6–16] and agrochemical research [16–20]. About 20% of all pharmaceuticals and roughly 40% of agrochemicals are fluorinated. Perfluoroalkyl substituents are particularly interesting as they often lead to a significant increase in lipophilicity and thus bioavailability albeit with a modified stability. Therefore, it is of continual interest to develop new, environmentally benign methods for the introduction of these groups into target molecules. Recent years have witnessed exciting developments in mild catalytic fluorination techniques. In contrast to carbon–carbon, carbon–oxygen and carbon–nitrogen bond formations, catalytic carbon–fluorine bond formation remained an unsolved challenge, mainly due to the high electronegativity of fluorine, its hydration and thus reduced nucleophilicity [21]. The importance of this developing research field is reflected by the various review articles which have been published dealing with transition metal mediated or catalyzed fluorination [22–24], difluoromethylation [24], and trifluoromethylation reactions [22–28].

The present review focuses on fundamental achievements in the field of transition metal-catalyzed mono-, di- and trifluoromethylation as well as trifluoromethylthiolation of sp² carbon atoms. We present the different developments according to the reaction-type and the nature of the transition metal.

### 1 Catalytic monofluoromethylation

Monofluoromethylated aromatics find application in various pharmaceutical [29–32] and agrochemical products [18].

Although numerous methods for the catalytic introduction of a trifluoromethyl group onto aryl moieties have been reported in the literature [27,33–41], the incorporation of partially fluorinated methyl groups is still underdeveloped [42–43]. In most cases transition metals have to be employed in stoichiometric amounts.

#### 1.1 Palladium catalysis

**Scheme 1:**
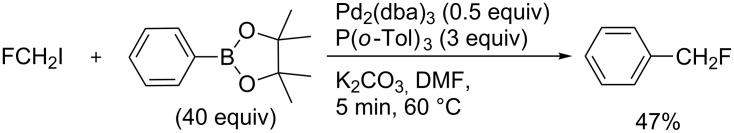
Pd-catalyzed monofluoromethylation of pinacol phenylboronate [44].

The first monofluoromethylation was reported by M. Suzuki (Scheme 1) [44]. Fluoromethyl iodide was reacted with pinacol phenylboronate (40 equiv) affording the coupling product in low yield (47%).

The Pd-catalyzed α-arylation of α-fluorocarbonyl compounds affording various quaternary α-aryl-α-fluorocarbonyl derivatives has been reported by J. F. Hartwig [45], J. M. Shreeve [46] and further investigated and generalized to both open-chain and cyclic α-fluoroketones by F. L. Qing [47–48]. However, further decarbonylation to the monofluoromethyl group proved difficult.

#### 1.2 Copper catalysis

**Scheme 2:**
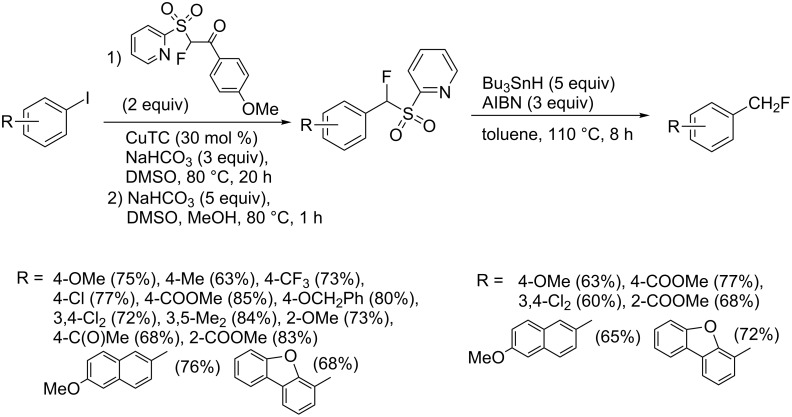
Cu-catalyzed monofluoromethylation with 2-PySO_2_CHFCOR followed by desulfonylation [49].

Recently a copper-catalyzed monofluoromethylation was described by J. Hu. Aryl iodides were submitted to a Cu-catalyzed (CuTC = copper thiophene-2-carboxylate) debenzoylative fluoroalkylation with 2-PySO_2_CHFCOR followed by desulfonylation (Scheme 2) [49]. It has been shown that the (2-pyridyl)sulfonyl moiety is important for the Cu-catalysis.

### 2 Catalytic difluoromethylation

The synthesis of difluoromethylated aromatics attracted considerable interest in recent years due to their potential pharmacological and agrochemical activity [42,50–56].

#### 2.1 Copper catalysis

**Scheme 3:**
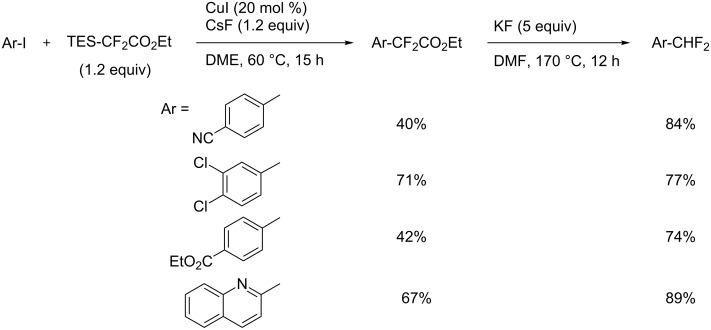
Cu-catalyzed difluoromethylation with α-silyldifluoroacetates [57].

In contrast to widely used stoichiometric copper-mediated trifluoromethylations and the recent results of the Cu-catalyzed reaction described above, that of difluoromethylation has been more slowly developed. This is probably due to the lack of thermal stability of CuCHF_2_ [42]. To the best of our knowledge, the direct cross-coupling of CuCHF_2_ with aromatic halides has not been reported. H. Amii reported on the reaction of aryl iodides with α-silyldifluoroacetates in the presence of a catalytic amount of CuI (Scheme 3). The corresponding aryldifluoroacetates have been obtained in moderate to good yields and afforded, after subsequent hydrolysis of the aryldifluoroacetates and KF-promoted decarboxylation, a variety of difluoromethyl aromatics [57].

Unlike previous protocols where an excess of copper is required, this approach presents some advantages such as: (i) stability and availability of the required 2-silyl-2,2-difluoroacetates from trifluoroacetates or chlorodifluoroacetates [58–60]; (ii) high functional group tolerance as the reactions proceed smoothly under mild conditions; and (iii) the reaction being catalytic in copper.

J. Hu described the Lewis acid (CuF_2_·2H_2_O) catalyzed vinylic C–CHF_2_ bond formation of α,β-unsaturated carboxylic acids through decarboxylative fluoroalkylation (Table 1) [61]. A wide range of α,β-unsaturated carboxylic acids afforded the corresponding difluoromethylated alkenes in high yields and with excellent *E*/*Z* selectivity.

**Table 1:** Cu-catalyzed C–CHF_2_ bond formation of α,β-unsaturated carboxylic acids through decarboxylative fluoroalkylation [61].

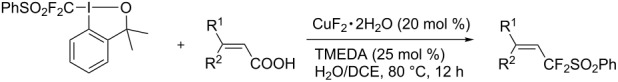					

Compound	Yield (%)	Compound	Yield (%)	Compound	Yield (%)

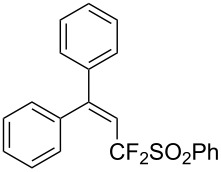	70	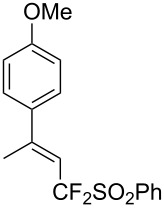	88	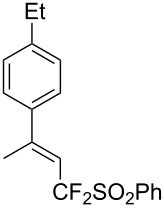	86
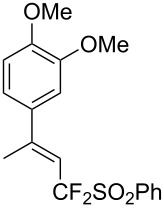	90	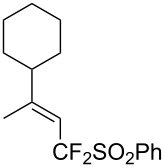	87	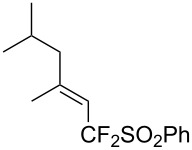	91
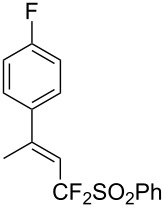	86	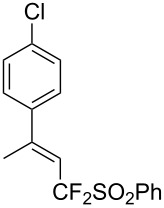	87	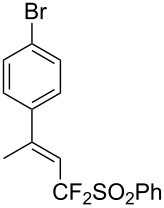	86
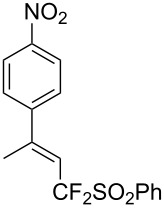	82	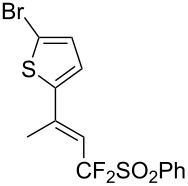	76	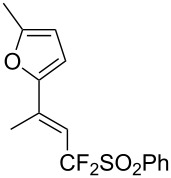	60
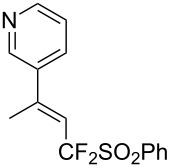	60	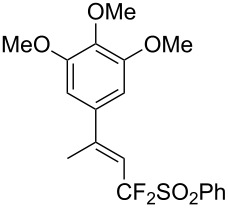	90	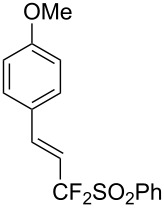	84
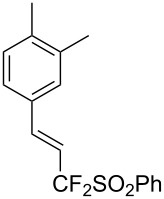	84	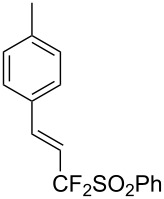	73	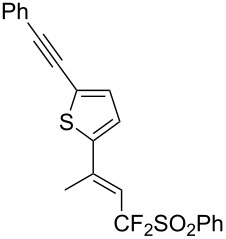	70
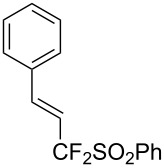	65	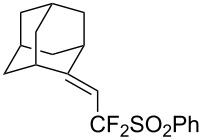	63		

**Figure 1:**
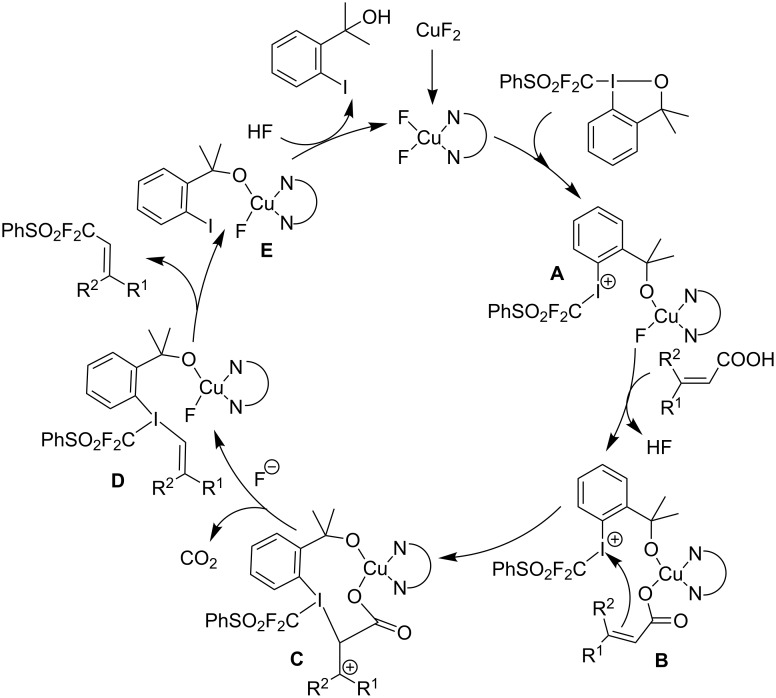
Mechanism of the Cu-catalyzed C–CHF_2_ bond formation of α,β-unsaturated carboxylic acids through decarboxylative fluoroalkylation [61].

The putative mechanism for this copper-catalyzed decarboxylative fluoro-alkylation involves the iodine–oxygen bond cleavage of Togni's reagent in presence of the copper catalyst to produce a highly electrophilic species (intermediate **A**). Then, the acrylate derivative coordinates to the iodonium salt **A** leading to intermediate **B** with generation of hydrogen fluoride, followed by an intramolecular reaction between the double bond and the iodonium ion to provide intermediate **C**. The presence of HF in the reaction medium promotes the decarboxylation step in intermediate **C**, and subsequent reductive elimination leads to the formation of the thermodynamically stable *E*-alkene. Finally, protonation of intermediate **E** regenerates the copper catalyst, thus allowing the catalytic turnover (Figure 1).

#### 2.2 Iron catalysis

**Scheme 4:**
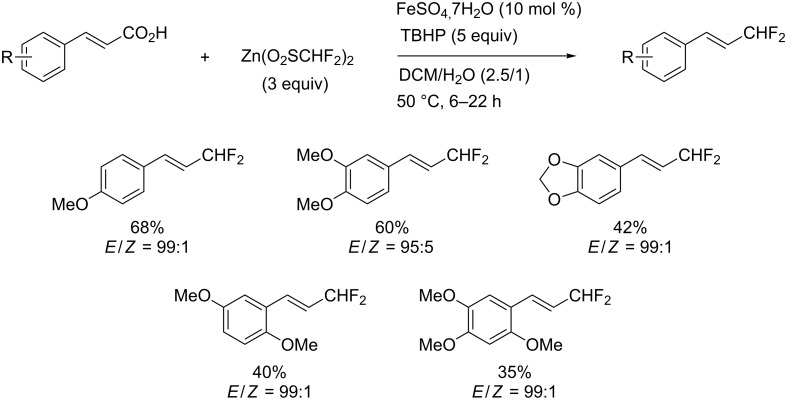
Fe-catalyzed decarboxylative difluoromethylation of cinnamic acids [62].

Similarly to the work of J. Hu and colleagues using copper catalysis, the group of Z.-Q. Liu reported on the decarboxylative difluoromethylation of α,β-unsaturated carboxylic acids. However, the latter used iron(II) sulfate as catalyst and zinc bis(difluoromethanesulfinate) as the fluoroalkyl transfer reagent. A handful of β-difluoromethylstyrenes were obtained in moderate yields and with complete diastereoselectivity (Scheme 4) [62].

### 3 Catalytic perfluoroalkylation

The transition metal mediated trifluoromethylation of aromatic compounds has been extensively reviewed in recent years by several authors [23–28,63–64]. Nevertheless, aromatic trifluoromethylations catalytic in metal are still rare. This section reviews recent advances in this area and classifies the reactions according to metal type and reaction mechanism. One can identify two major approaches, trifluoromethylation via cross-coupling reactions or the more recent C–H functionalization.

#### 3.1 Palladium catalysis

**3.1.1 Trifluoromethylation of Csp^2^–X bonds (X = halogen or sulfonate) by means of a nucleophilic CF_3_-source.** The first Pd-catalyzed aromatic trifluoromethylation of aryl chlorides with a nucleophilic source of CF_3_ has been reported in 2010 by S. L. Buchwald et al. (Table 2) [38]. An excess of expensive (trifluoromethyl)triethylsilane (TESCF_3_) in combination with potassium fluoride was used to provide the expected trifluoromethylated arenes in good yields, and a variety of functional groups is tolerated under the mild conditions of the process. The reaction with aryl bromides or triflates is less efficient. The success of this Pd-catalyzed trifluoromethylation is due to highly hindered phosphorus ligands like BrettPhos, which facilitate the reductive elimination step. However, the phosphine was changed for the less bulky ligand RuPhos for the reaction with *ortho*-substituted aryl chlorides. The authors presume a Pd(0)/Pd(II) catalytic cycle, which is supported by preliminary mechanistic studies.

**Table 2:** Pd-catalyzed trifluoromethylation of aryl and heteroaryl chlorides [38].

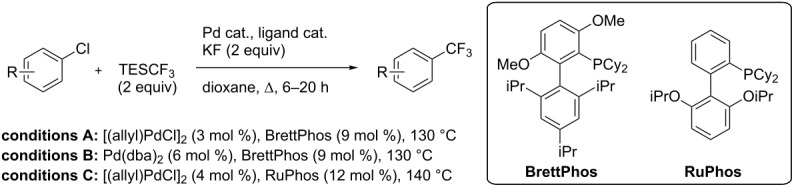					

Compound	Conditions	Yield (%)	Compound	Conditions	Yield (%)

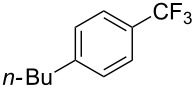	A	80	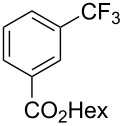	A	83
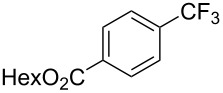	A	85	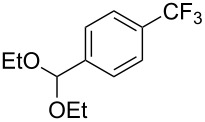	A	72
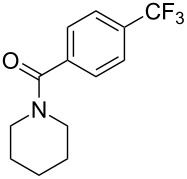	A	94	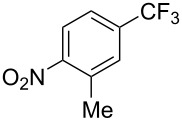	A	70
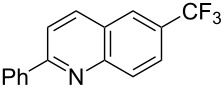	A	82	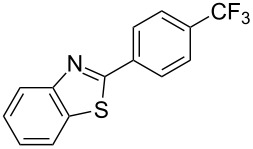	A	90
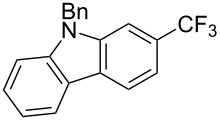	A	76	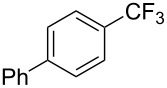	A	84
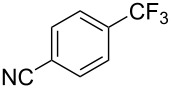	B	72	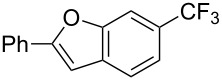	B	87
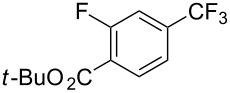	B	72	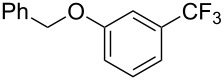	B	88
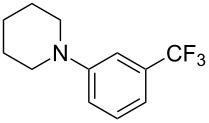	B	84	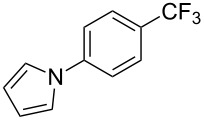	B	84
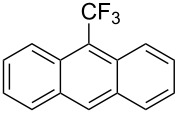	C	90	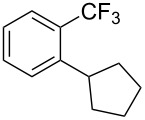	C	77
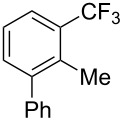	C	87	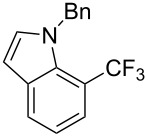	C	78

In 2011, B. S. Samant and G. W. Kabalka developed improved conditions for the trifluoromethylation of aryl halides by carrying out the reaction in sodium dodecyl sulfate (SDS) and toluene, and by using TMSCF_3_ as a cheaper trifluoromethylating agent [65]. The reverse micelles appear to prevent the decomposition of TMSCF_3_ and provide an effective reaction site for oxidative addition of Ar–X and the Pd(0) catalyst, increasing the yields and allowing the use of aryl bromides as starting materials (Table 3). Free alcohols and amines are compatible with the reaction conditions, which was not the case with S. L. Buchwald’s methodology.

**Table 3:** Pd-catalyzed trifluoromethylation of bromoaromatic compounds in micellar conditions [65].

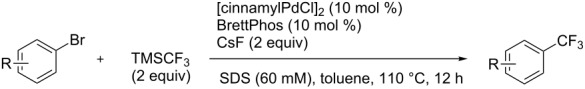					

Compound	Yield (%)	Compound	Yield (%)	Compound	Yield (%)

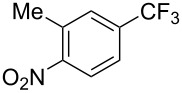	77	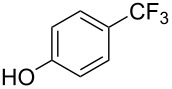	70	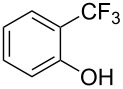	74
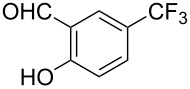	68	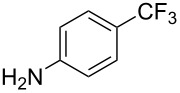	71	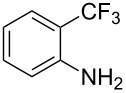	70
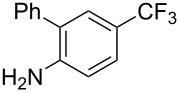	72	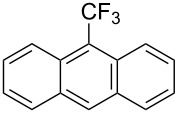	80		

For the metal-catalyzed perfluoroalkylation of sp^2^ carbons, vinyl sulfonates represent valuable alternative coupling partners to vinyl halides, given that they can be prepared in a straightforward manner from readily available alcoholic precursors. In 2011, the group of S. L. Buchwald described a catalytic system to convert cyclic vinyl triflates or nonaflates to their trifluoromethylated equivalents (Table 4) [66]. Ruppert’s reagent was used as the CF_3_^–^ precursor in a combination with potassium fluoride as an activator for the reaction with vinyl triflates, while TESCF_3_ and rubidium fluoride gave better results for nonaflate electrophiles. Otherwise, the scope is actually limited to six-membered vinyl sulfonates, and moderate yields were obtained with 2-alkyl substituted cyclohexenyl substrates.

**Table 4:** Pd-catalyzed trifluoromethylation of vinyl sulfonates [66].

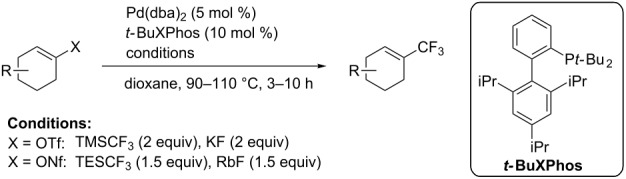					

Compound	X =	Yield (%)	Compound	X =	Yield (%)

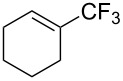	OTf	83	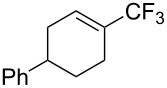	OTf	81
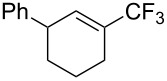	OTf	62	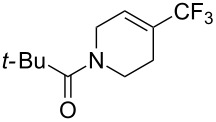	OTf	53
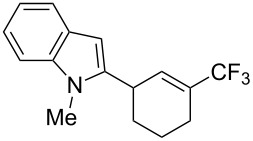	OTf	84	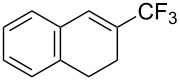	OTf	75^a^
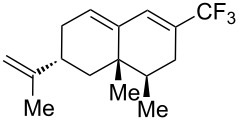	OTf	74^a^	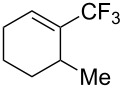	OTf	40
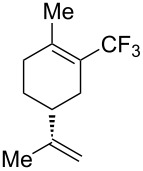	OTf	36^a^	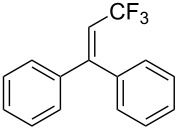	OTf	71^a^
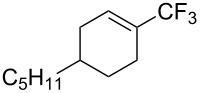	ONf	73^a^	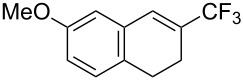	ONf	80^a^
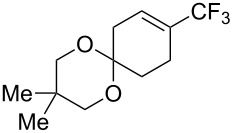	ONf	51			

^a^[(allyl)PdCl]_2_ was used instead of Pd(dba)_2_.

**3.1.2 Trifluoromethylation by means of C–H activation and an electrophilic CF_3_-source.** In 2010, J.-Q. Yu and coworkers reported on the first Pd-catalyzed trifluoromethylation at C–H positions in aromatic compounds (Table 5) [67]. Pd(OAc)_2_ (10 mol %) was used as the catalyst, and Umemoto’s sulfonium tetrafluoroborate salt as the CF_3_ source rather than its triflate analogue. Trifluoroacetic acid and copper(II) acetate as additives proved essential for achieving high yields of the desired trifluoromethylated arenes. 2-Arylpyridines, but also other aryl-substituted heteroarenes were successfully trifluoromethylated with complete regioselectivity in the position *ortho* to the aryl–heteroaryl bond, with moderate to high yields in most cases. Obviously, the heteroaryl group served as a directing group in this transformation. Interestingly, all isomers of 2-tolylpyridine were trifluoromethylated with highest yields; while in the case of chloro or methoxy groups, the efficiency of the reaction was dependent on the position of the substituent relative to the heteroaryl group. Notably, the chloro-substituted substrates required higher catalyst loadings for sufficient conversion. The authors also note that keto, ester and nitro substituents led to poor yields. The mechanism of this transformation and the role of the additives have not been elucidated yet.

**Table 5:** Pd-catalyzed C–H trifluoromethylation employing Umemoto’s sulfonium tetrafluoroborate salt [67].

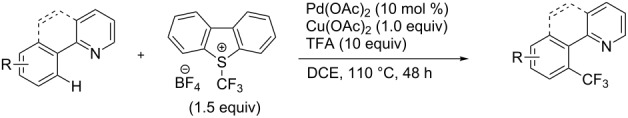				

Product		Yield (%)^a^	Product	Yield (%)^a^

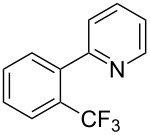		86	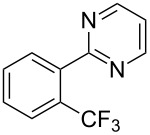	0^c^
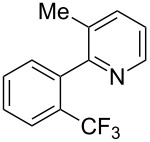		82	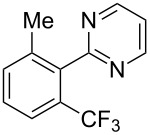	88
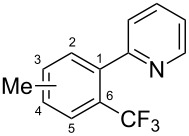	2-Me 3-Me 4-Me	84 83 83	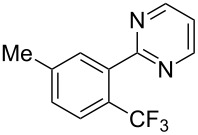	75^c^
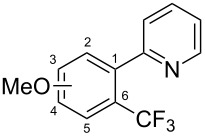	2-OMe 3-OMe 4-OMe	78 54^b^ 68	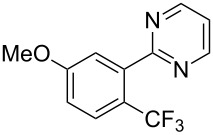	58^c^
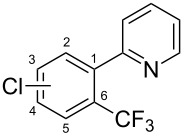	2-Cl 3-Cl 4-Cl	55^c^ 75^c^ 72^c^	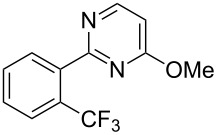	62^c^
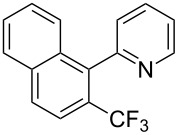		78^b^	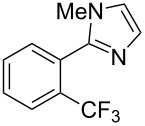	53^c^
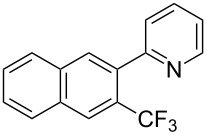		87^b^	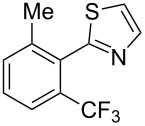	74
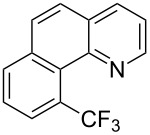		88		

^a^Yields for isolated compounds. ^b^15 mol % of Pd(OAc)_2_ were used. ^c^20 mol % of Pd(OAc)_2_ were used.

**Scheme 5:**
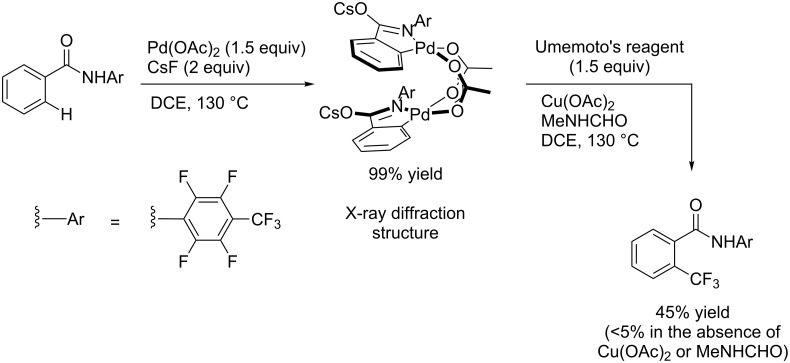
Preliminary experiments for investigation of the mechanism of the C–H trifluoromethylation of *N*-arylbenzamides [68].

The group of J.-Q. Yu further studied this reaction by adapting it to secondary *N*-arylbenzamides as more versatile substrates than arylpyridines [68]. In comparison with the previous reaction conditions, two equivalents of Cu(OAc)_2_ had to be used instead of one, and *N*-methylformamide as an additive appeared essential. On the other hand, the counteranion of sulfonium in Umemoto’s reagent had no influence on the reaction. Variously substituted arenes underwent trifluoromethylation with moderate to excellent yields (Table 6). Interestingly, bromo-, chloro- or ester-substituted substrates were also converted, allowing further derivatization. As a preliminary investigation on the mechanism of the reaction, the authors prepared an analogue of the palladacyclic intermediate supposed to be involved in the first stages of the catalytic cycle and submitted it to the reaction conditions, in the presence or not of the amide additive and of Cu(OAc)_2_ (Scheme 5). These results confirmed the indispensable involvement of these additives in the mechanism.

**Table 6:** Extension of Yu’s C–H trifluoromethylation to *N*-arylbenzamides [68].

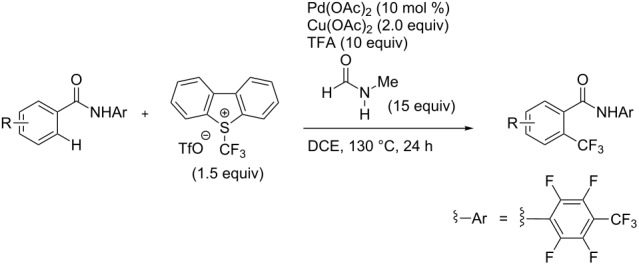				

Product		Yield (%)^a^	Product	Yield (%)^a^

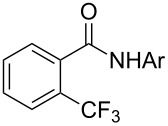		79	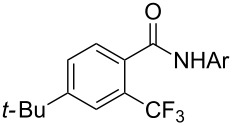	77
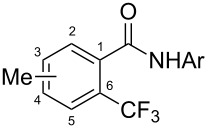	2-Me 3-Me 4-Me	84 94 53	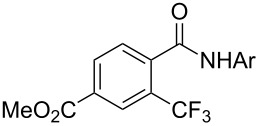	55
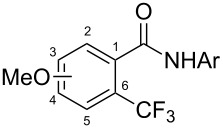	3-OMe 4-OMe	89 56	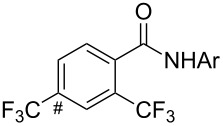	32^b^
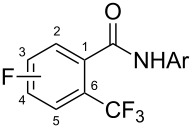	3-F 4-F	56 61	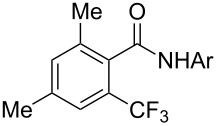	71
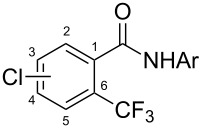	2-Cl 3-Cl 4-Cl	41 81 40	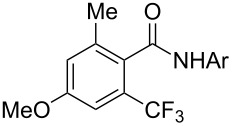	72
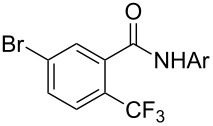		82	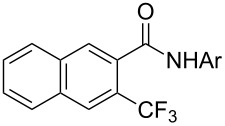	75
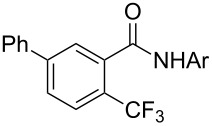		67		

^a^Yields for isolated compounds. ^b^2 equiv of Umemoto’s reagent were used for 48 h. ^#^Indicates the initial CF_3_ substituent present in the substrate.

**Figure 2:**
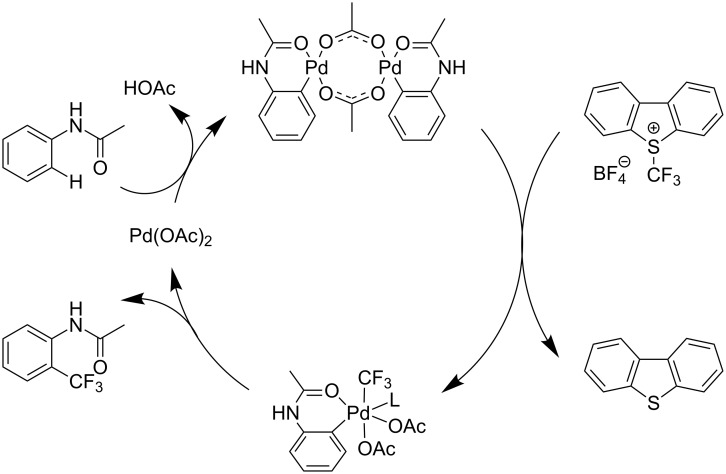
Plausible catalytic cycle proposed by Z.-J. Shi et al. for the trifluoromethylation of acetanilides [69].

A complementary study by Z.-J. Shi and coworkers investigated the trifluoromethylation of acetanilides also using palladium(II) and copper(II) acetates as catalyst and additive respectively, with Umemoto’s reagent [69]. Pivalic acid (vs TFA in the case of J.-Q. Yu et al.) as an additive gave the best results. Diversely functionalized substrates were converted to the corresponding benzotrifluorides with up to 83% yield (Table 7). Striking features of the reaction were the ability to use alkoxycarbonyl-, benzoyl, acetyl- and acetoxy-substituted acetanilides, and, above all, halogenated arenes including fluoro-, chloro-, bromo- and iodoacetanilides, rendering further functionalization possible. However, the presence of a methoxy or trifluoromethoxy group *meta* to the directing group shuts down the reaction completely. Other directing groups were investigated. When hydrogen was replaced by methyl on nitrogen in the starting acetanilide, no reaction occurred; on the other hand, *N*-pivaloyl- and *N*-benzoylanilines were trifluoromethylated, albeit with lower yields than acetanilide. From the study of kinetic isotope effects in several experiments as well as of a Pd-insertion complex similarly to the work of J.-Q. Yu et al., the authors proposed a Pd(II)/Pd(IV) catalytic cycle starting with C–H activation of the substrate followed by oxidation of the complex with Umemoto’s reagent and completed by reductive elimination of the desired benzotrifluoride (Figure 2).

**Table 7:** Shi’s C–H trifluoromethylation of acetanilides [69].

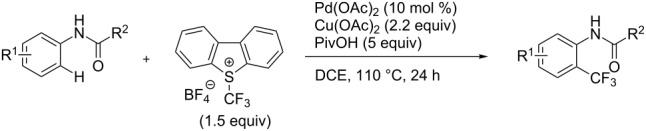					

Product		Yield (%)^a^	Product		Yield (%)^a^

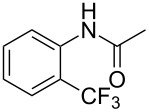		69	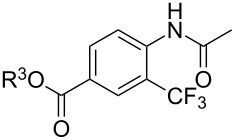	R^3^ = Me R^3^ = Et	64 83
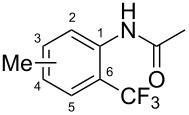	2-Me 3-Me 4-Me	51 47 63	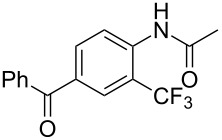		72
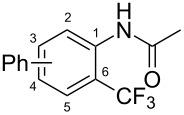	3-Ph 4-Ph	66 46	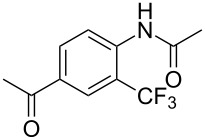		41
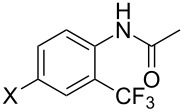	F Cl Br I	71 72 66 48	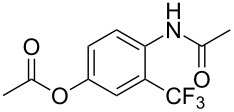		56
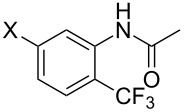	F Cl Br	52 53 63	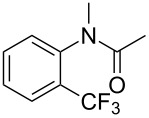		0
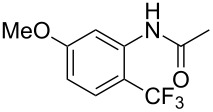		0	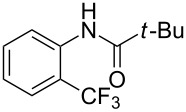		41
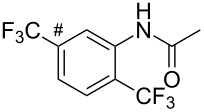		Trace	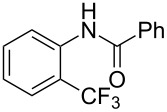		42
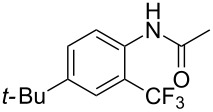		77			

^a^Yields for isolated compounds. ^b^2 equiv of Umemoto’s reagent were used for 48 h. ^#^Indicates the initial CF_3_ substituent present in the substrate.

**Figure 3:**
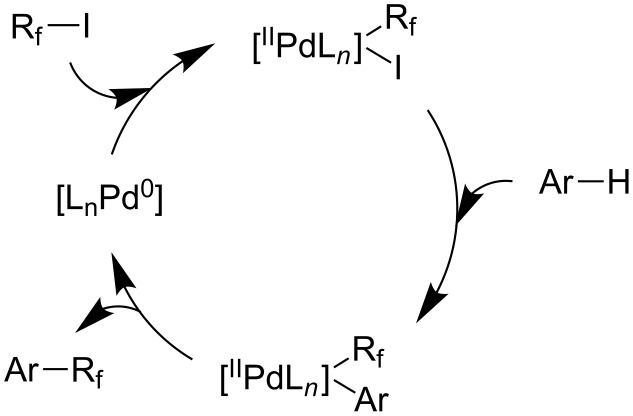
Plausible catalytic cycle proposed by M. S. Sanford et al. for the perfluoroalkylation of simple arenes using perfluoroalkyl iodides [70].

**3.1.3 Perfluoroalkylation by means of C–H activation and a perfluoroalkyl radical-source.** In contrast to the studies described above, the group of M. S. Sanford has developed a Pd-catalyzed perfluoroalkylation of arenes in the absence of directing groups [70]. Perfluoroalkyl iodides were used as the source of the fluorinated alkyl group. Under the optimized reaction conditions, a mixture of the iodide, 5 mol % Pd_2_dba_3_, 20 mol % BINAP, cesium carbonate (2 equiv) and the arene (large excess) were heated under air in the absence of a cosolvent (Table 8). Benzene, naphthalene and several disubstituted benzenes were successfully transformed with 39–99% NMR yields and 27–76% isolated yields (relative to the starting perfluoroalkyl iodide). *N*-Methylpyrrole was also perfluoroalkylated in high yield. The reaction proved very selective in several aspects, since 1,2- and 1,3-disubstituted benzenes were all preferentially functionalized at the 4-position; aryl C–H positions were perfluoroalkylated but not benzylic positions; and only the 2-position in *N*-methylpyrrole was functionalized. A tentative mechanism was proposed, based on the literature on each of the assumed steps of the catalytic cycle (Figure 3). After oxidative addition of the perfluoroalkyl iodide onto palladium(0), the iodide ligand is replaced by aryl by C–H activation, and a reductive elimination of the desired product liberates the palladium catalyst. Experiments carried out by the authors were inconsistent with an alternative purely free radical pathway, but could not rule out caged and/or “Pd-associated” radical intermediates.

**Table 8:** Sanford’s Pd-catalyzed perfluoroalkylation at a C–H position of (hetero)arenes in the absence of directing groups [70].

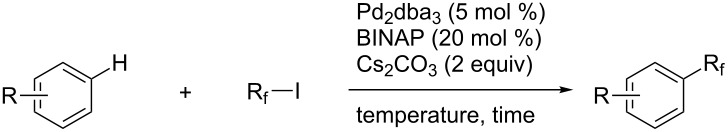					

Product (isomer ratio)	Temp., Time	NMR (and isolated) yields (%)	Product (isomer ratio)	Temp., Time	NMR (and isolated) yields (%)

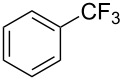 (---)	100 °C, 15 h	26^a^	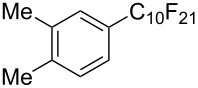 (>20:1)	100 °C, 15 h	76 (54)
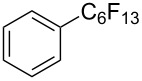 (---)	80 °C, 15 h	81^a^	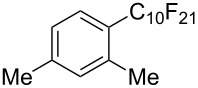 (2.2:1:0)	60 °C, 24 h	77 (55)
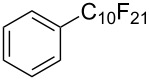 (---)	80 °C, 15 h	79 (60)	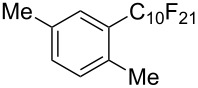 (---)	60 °C, 24 h	52 (52)
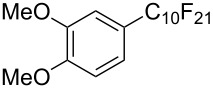 (>20:1)	80 °C, 15 h	79 (76)	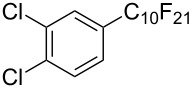 (>20:1)	100 °C, 15 h	39 (27)
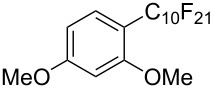 (17:1:2)	100 °C, 15 h	99 (69)	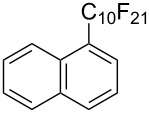 (4.0:1)	100 °C, 15 h	76 (34)
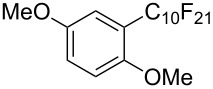 (---)	100 °C, 15 h	84 (59)	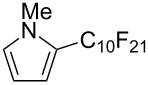 (>20:1)	40 °C, 15 h	99 (70)
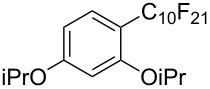 (11:1:1)	80 °C, 15 h	80(69)			

^a^GC yield (%).

Another study by Y. H. Budnikova et al. described the electrochemical perfluoroalkylation of 2-phenylpyridine in the presence of palladium(II) catalysts (10 mol %) and starting either from 6*H*-perfluorohexyl bromide or perfluoroheptanoic acid [71]. Interestingly, the latter reagent provided the highest yields, and the reaction appeared to proceed through an intermediate biaryl perfluoroalkylcarboxylate, which extrudes CO_2_ to yield the desired product (Table 9). As underlined by the authors, the electrocatalytic reactions proceed under mild conditions at potentials that clearly generate high oxidation state metals.

**Table 9:** Pd-catalyzed electrochemical perfluoroalkylation of 2-phenylpyridine [71].

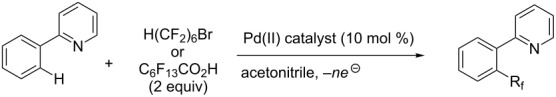				

Perfluoroalkyl source	Pd(II) catalyst			
	Pd(OAc)_2_	Yield (%)	Pd_2_(*o*-C_6_H_4_Py)_2_(OAc)_2_	Yield (%)

H(CF_2_)_6_Br	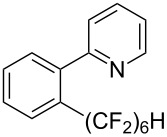	10	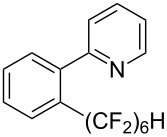	30
C_6_F_13_CO_2_H	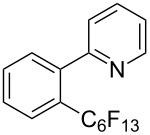	≤18	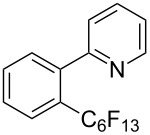	81
	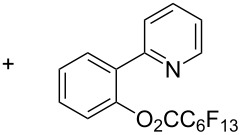			

**3.1.4 Trifluoromethylation by means of presumed C–H activation and a nucleophilic CF_3_-source.** A single study on palladium-catalyzed trifluoromethylation of sp^2^-C–H bonds was reported by G. Liu and coworkers [72]. It described the introduction of a CF_3_ group at the 2-position of indoles using palladium acetate as a catalyst and the Ruppert–Prakash reagent TMSCF_3_. A screening of reaction conditions showed that cesium fluoride proved the best base. PhI(OAc)_2_ was the preferred oxidant over other hypervalent iodine compounds or sources of F^+^ or CF_3_^+^; additionally, the presence of a bis(oxazoline) as a ligand was beneficial to the reaction, as well as that of TEMPO to prevent trifluoromethylation of the benzene ring as a side reaction. With these optimized reaction conditions, a series of indoles was successfully trifluoromethylated (Table 10). The nature of the substituent on nitrogen had a strong influence on yields. Alkyl or alkyl-derived groups as well as phenyl gave moderate to good results, but *N*-tosyl or *N*–H gave almost no desired product, if any. Indoles bearing substituents at the 2 or 3 positions were suitable substrates for respective 3- or 2-functionalization, although an ester group in position 3 led to a lower yield; a “naked” indole ring could be trifluoromethylated in a 39% yield. Electron-donating or -withdrawing groups on the benzo moiety were tolerated, and in particular, the presence of a halogen atom in position 5 gave yields almost as high as in the case of the unsubstituted analogue. By comparing the activities in the case of substrates bearing electron-donating and -releasing groups at the 5-position, and considering the regioselective 3-functionalization of *N*-methylindole, the authors proposed the following catalytic cycle: 1) electrophilic palladation of indole, 2) oxidation of the resulting Pd(II) species by the combination of the hypervalent iodine reagent and TMSCF_3_ to give a CF_3_-Pd(IV) intermediate, and 3) reductive elimination leading to the desired trifluoromethylindole.

**Table 10:** Pd-catalyzed trifluoromethylation of sp^2^-C–H bonds of indoles employing TMSCF_3_ [72].

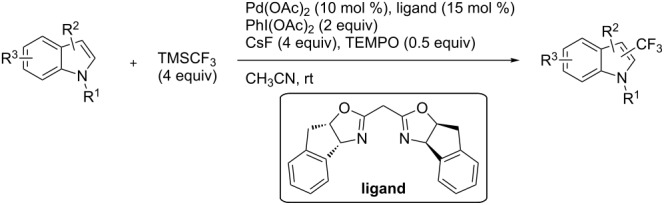					

Product		Yield (%)^a^	Product		Yield (%)^a^

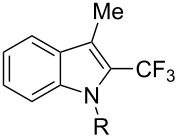	Me Et Bn *n-*Bu Ph SEM^b^ Ts H	83 72 62 63 50 57 <5 0	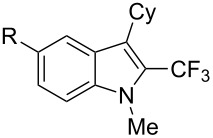	Me OMe Cl Br E^c^	60 56 67 70 51

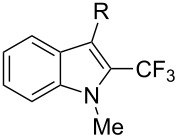	Cy *c-*C_5_H_9_ iPr (CH_2_)_2_OMe CH_2_CHE_2_^c^ E^c^	75 71 61 70 66 33	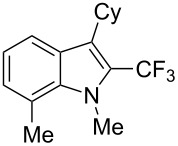		60

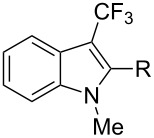	Me Ph	65 66	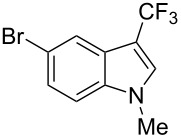		39

^a^Isolated yields. ^b^SEM = TMS(CH_2_)_2_OCH_2_. ^c^E = CO_2_Me.

#### 3.2 Copper catalysis

**3.2.1 Trifluoromethylation of Csp^2^–X bonds (X = halogen) by means of a nucleophilic CF_3_-source.** In 2009, H. Amii et al. reported on the first general copper-catalyzed trifluoromethylation of aryl iodides with TESCF_3_ in presence of potassium fluoride [33]. After activation of the fluoroalkylsilane by the fluoride, the trifluoromethyl anion is generated and leads to the formation of the CF_3_Cu species. Then, σ-bond metathesis between Ar–I and CF_3_–Cu yields trifluoromethylated arenes with regeneration of CuI. To perform the reaction catalytically, the use of a diamine ligand was necessary to enhance the electron density at the metal center, thus increasing the rate of σ-bond metathesis. In this way, the copper catalyst is regenerated faster and avoids in situ decomposition of the CF_3_^−^ species. Heteroaromatic iodides and iodobenzenes bearing electron-withdrawing groups participated smoothly in cross-coupling reactions with good yields (Table 11).

**Table 11:** The first Cu-catalyzed trifluoromethylation of aryl iodides [33].

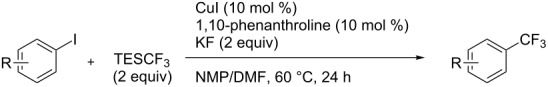					

Compound	Yield (%)^a^	Compound	Yield (%)^a^	Compound	Yield (%)^a^

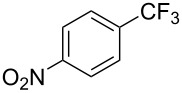	90	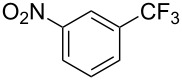	90	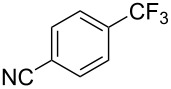	80
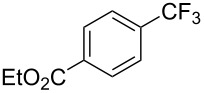	89	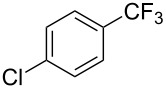	63	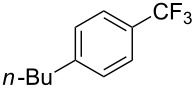	44
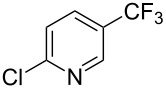	69	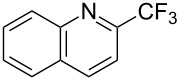	99	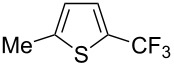	63

^a^NMR yield calculated by ^19^F NMR by using 2,2,2-trifluoroethanol as an internal standard.

**Figure 4:**
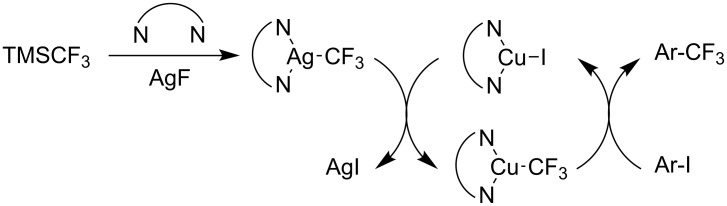
Postulated reaction pathway for the Ag/Cu-catalyzed trifluoromethylation of aryl iodides by Z. Q. Weng et al. [73].

Later, modified conditions were proposed by Z. Q. Weng et al. where *N,N’*-dimethylethylenediamine (DMEDA) and AgF were used instead of 1,10-phenanthroline and KF respectively [73]. In addition to activating the silyl group of the trifluoromethylating agent, the silver salt also acts as a stabilizer for the CF_3_^−^ species and prevents its self-decomposition (Figure 4). As a result, the more economical TMSCF_3_ can be employed, and good yields were observed for both electron-rich and electron-poor aryl iodides in this cooperative silver-assisted copper-catalyzed trifluoromethylation (Table 12).

**Table 12:** Cooperative effect of silver for the copper-catalyzed trifluoromethylation of aryl iodides [73].

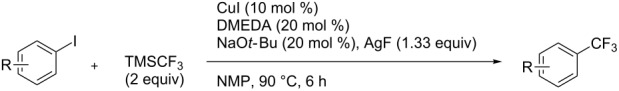					

Compound	Yield (%)	Compound	Yield (%)	Compound	Yield (%)

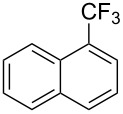	75^b^	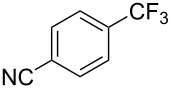	89	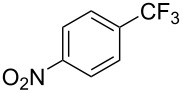	98^b^
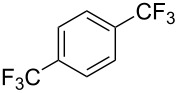	64	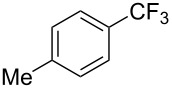	73	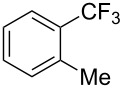	59
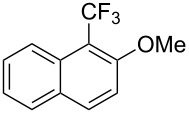	47	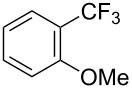	66	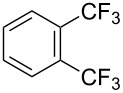	61
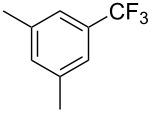	76^b^				

^a^NMR yield calculated by ^19^F NMR by using hexafluorobenzene as an internal standard. ^b^Isolated yield.

Even if the pioneering work of H. Amii and Z. Q. Weng resulted in the development of reliable and robust catalytic systems, they suffer from the lack of accessibility to inexpensive, stable and easy-to-handle reagents that could be used as convenient CF_3_ sources for nucleophilic trifluoromethylations. The group of L. J. Gooßen et al. was the first to propose a new crystalline, air-stable (trifluoromethyl)trimethoxyborate as an alternative to Ruppert’s reagent [74]. This innovative reagent is readily accessible by reaction of TMSCF_3_ with B(OMe)_3_ and KF in THF, and allows the conversion of a broad scope of aryl iodides in high yields without the need for basic additives (Table 13).

**Table 13:** Cu-catalyzed trifluoromethylation of (hetero)aryl iodides with (trifluoromethyl)trimethoxyborate [74].

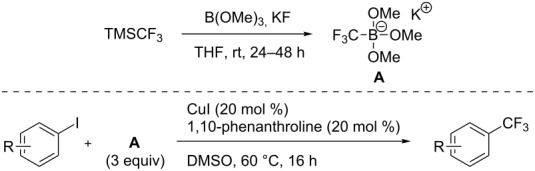					

Compound	Yield (%)	Compound	Yield (%)	Compound	Yield (%)

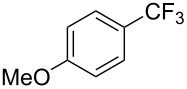	77	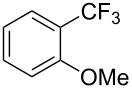	83	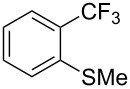	91
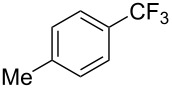	74	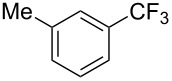	92	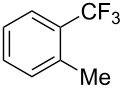	70
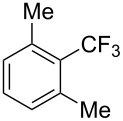	59	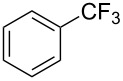	91	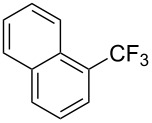	97
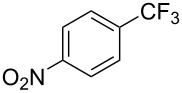	81	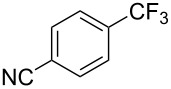	95	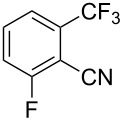	76
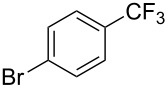	93	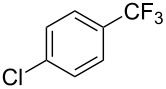	75	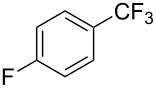	81
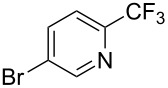	82	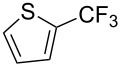	85	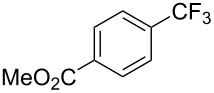	84
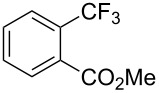	96	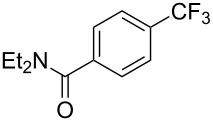	95	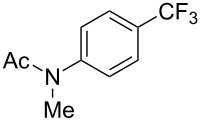	96
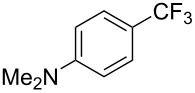	52	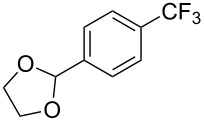	84		

Hemiaminals of trifluoroacetaldehyde are also considered to be convenient sources of trifluoromethyl anion [75]. H. Amii et al. reported on the use of an *O*-silylated hemiaminal as a cross-coupling partner for aromatic trifluoromethylation with a copper iodide/1,10-phenanthroline catalytic system [76]. Compound **B** was prepared from commercially available hemiacetal of fluoral and morpholine, following the procedure described by B. R. Langlois et al. [77] Moderate to good yields were observed when the reaction was carried out in diglyme with cesium fluoride as a base (Table 14).

**Table 14:** Cu-catalyzed trifluoromethylation of (hetero)aryl iodides with *O*-silylated hemiaminal of fluoral [76].

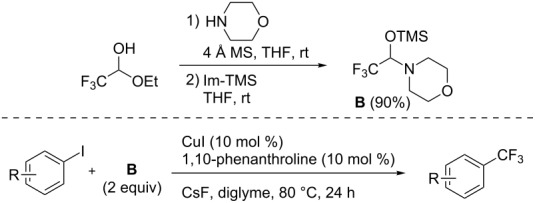					

Compound	Yield (%)^a^	Compound	Yield (%)^a^	Compound	Yield (%)^a^

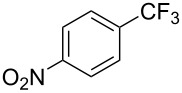	77	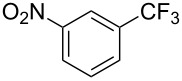	90	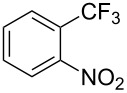	47
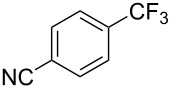	93	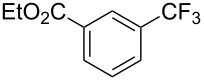	60	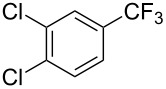	97
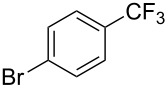	53	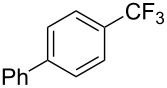	53	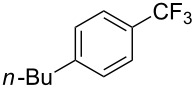	40
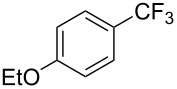	57	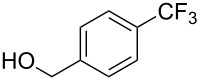	44	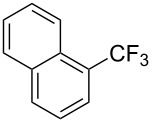	97
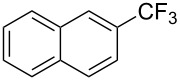	95	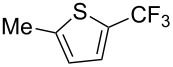	75		

^a^NMR yield calculated by ^19^F NMR by using trifluoromethoxybenzene as an internal standard.

**Figure 5:**
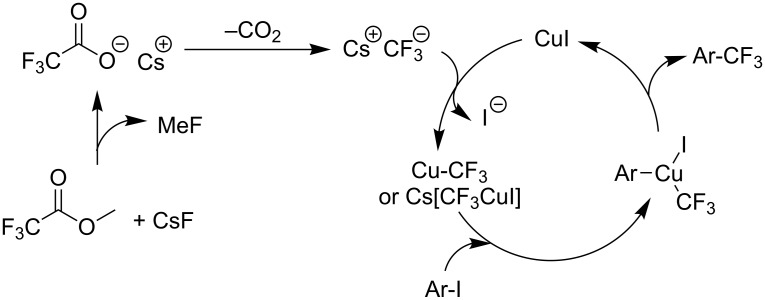
Postulated reaction mechanism for Cu-catalyzed trifluoromethylation reaction using MTFA as trifluoromethylating agent [81].

More recently, compounds derived from trifluoroacetic acid appeared to be a cheap and readily available nucleophilic trifluoromethyl source after decarboxylation at high temperature in the presence of stoichiometric amounts of copper salts [78–79]. In 2011, Y. M. Li et al. showed that the Cu-catalyzed C–CF_3_ bond formation of iodoarenes could be achieved by using a sodium salt of trifluoroacetic acid as the source of CF_3_^−^ [80]. Ag_2_O was chosen as an additive to promote the decarboxylation, and to accelerate the reductive elimination step by precipitation of AgI. To circumvent the use of moisture-sensitive sodium trifluoroacetate, M. Beller et al. employed a combination of methyl trifluoroacetate (MTFA) and cesium fluoride to generate the trifluoroacetate anion which decarboxylated under the reaction conditions (Figure 5). In most cases, the system does not necessitate the use of amine ligands excepted when aryl bromides are used instead of aryl iodides [81]. Aryl and heteroaryl products were formed in good to excellent yields with a good functional group tolerance (Table 15).

**Table 15:** Cu-catalyzed trifluoromethylation of (hetero)aryl iodides and aryl bromides with methyl trifluoroacetate [81].

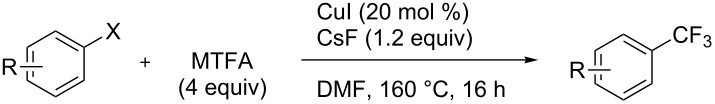					

Compound	X =	Yield (%)^a^	Compound	X =	Yield (%)^a^

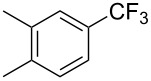	I	84	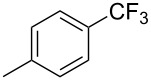	I	93
	Br	60^b,c^		Br	61^b,d^
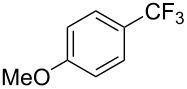	I	84	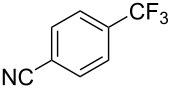	I	88
	Br	65^b,d^			47
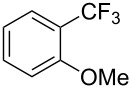	Br	62^b,c^	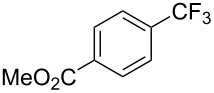	I	78
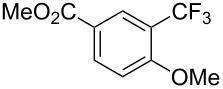	I	84^b,d^	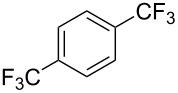	I	69
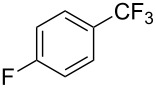	I	66	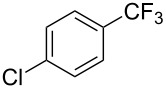	I	92
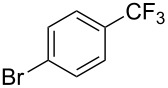	I	91	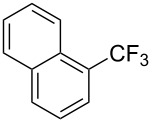	I	80
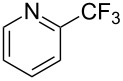	Br	50^b^	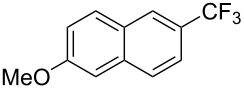	Br	95^c^

^a^NMR yield calculated by GC using tetradecane as an internal standard, ^b^20 mol % of 1,10-phenanthroline were added, ^c^CsF replaced by CsTFA, ^d^CsF replaced by CsCl.

**3.2.2 Trifluoromethylation of Csp^2^–H bonds by means of an electrophilic CF_3_-source.** In this section, the studies that are highlighted are distinguished by the nature of the substrates that are submitted to trifluoromethylation; indeed, all of them used the same electrophilic CF_3_ source, namely Togni’s benziodoxolone reagent.

M. Sodeoka and coworkers reported on the trifluoromethylation of indoles with Togni’s hypervalent iodine reagent in the presence of catalytic copper(II) acetate [82]. No additives were necessary, and this simple procedure allowed for the functionalization of various *N*–H as well as variously *N*-protected indoles with almost complete selectivity for the 2-position, even in the case of “naked” indoles (Table 16).

**Table 16:** Sodeoka’s trifluoromethylation of indoles with Togni’s hypervalent iodine reagent [82].

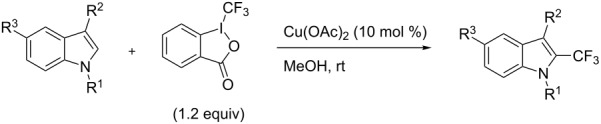			

Product		Isolated yield (%) (Time)	Yield based on recovered starting material (%)

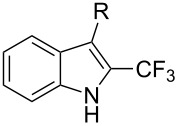	Me CO_2_Me	79 (6 h) 28 (24 h)	95 58

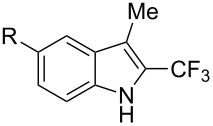	OMe Br	72 (18 h) 74 (24 h)	88 90

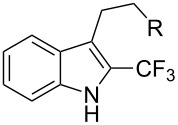	CO_2_Me NHBoc NHAc	72 (24 h) 68 (24 h) 79 (24 h)	79 76 93

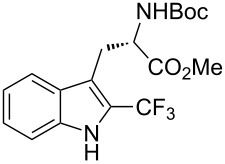		48 (24 h)	86

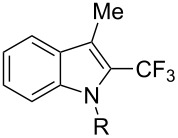	Me Bn Ac Boc	90 (6 h) 67 (18) 5 (24) 39 (24)	95 85 16 60

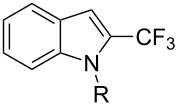	Me Bn	58 (6 h)^a^ 58 (6 h)	62^a^ 76

^a^Reaction carried out at 50 °C.

**Scheme 6:**
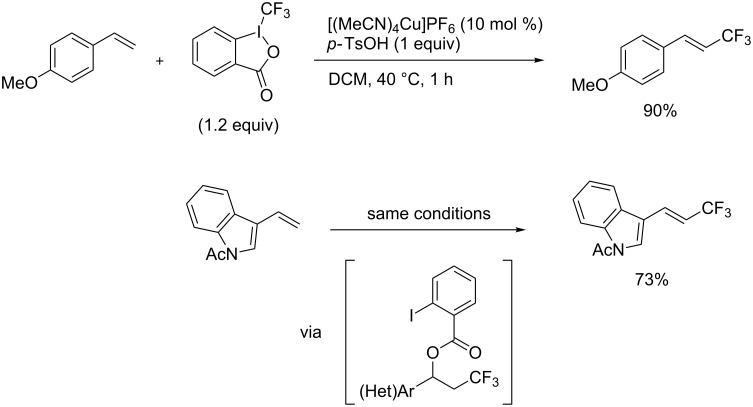
Formal Heck-type trifluoromethylation of vinyl(het)arenes by M. Sodeoka et al. [83].

The same group also reported on two examples of Heck-type copper-catalyzed trifluoromethylation of vinyl(het)arenes at the terminal carbon [83]. The reaction actually proceeded by oxytrifluoromethylation of the vinyl group, followed by elimination of the oxygen-leaving group in the presence of *p*-toluenesulfonic acid (Scheme 6).

**Figure 6:**
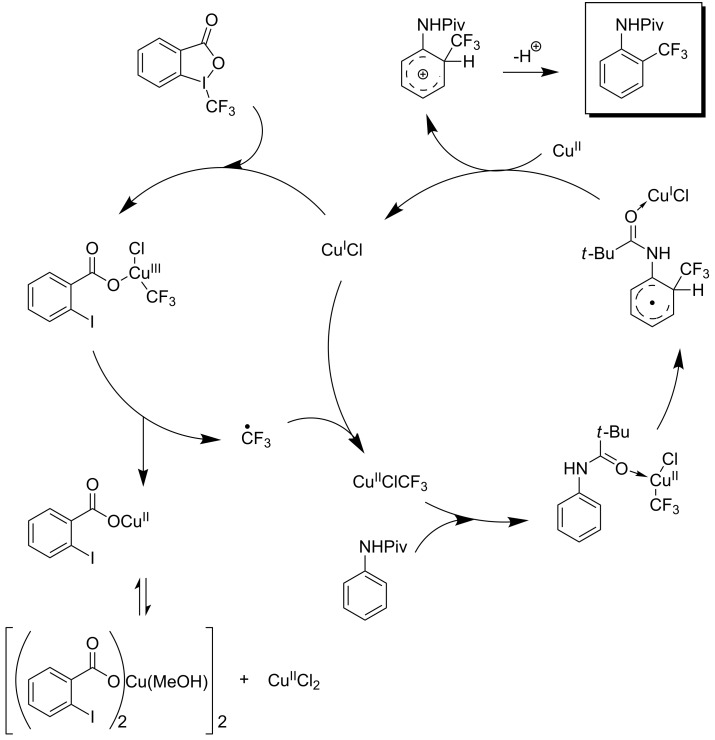
Proposed catalytic cycle for the copper-catalyzed trifluoromethylation of (het)arenes in presence of a pivalamido group (C. Chen, C. Xi et al.) [84].

Similarly to the Pd-catalyzed C–H trifluoromethylation of acetanilides by Z.-J. Shi et al., a copper-catalyzed process was developed by C. Chen and C. Xi and colleagues for the functionalization of pivanilides [84]. The latter methodology is simpler and more atom-economical since it does not require additives such as PivOH or stoichiometric metal salts as oxidants. However, it necessitates higher catalyst loadings (20 mol % CuCl vs 10 mol % Pd(OAc)_2_) to ensure acceptable yields. Various *N*-aryl and *N*-hetarylpivalamides were successfully converted under a nitrogen atmosphere, with introduction of the CF_3_ group predominantly *ortho* to the amide function (Table 17). Unlike the Pd-catalyzed reaction, this copper-catalyzed variant leads to a mixture of *ortho*-, *meta*- and *para*-functionalized compounds, with *ortho* > *para* > *meta* as the preferred order of selectivity in the case of simple pivanilide. Moreover, additional experiments in the presence of TEMPO or phenyl *N*-*tert*-butylnitrone (PBN) resulted respectively in no reaction and observation of the adduct of the CF_3_ radical on PBN by Electron Paramagnetic Resonance (EPR). These findings suggest a radical pathway for the mechanism of this reaction, as proposed by the authors and depicted in Figure 6.

**Table 17:** Cu-catalyzed C–H functionalization of pivanilides [84].

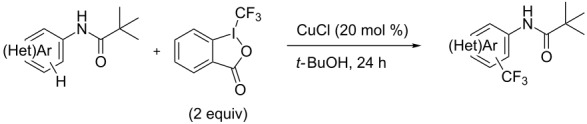				

Product		Temp. (°C)	Conversion (%)	Isolated yield (%) (NMR yield (%))

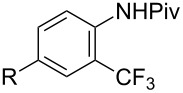	H Me iPr OMe F Cl Br CO_2_Et^a^	30 60 90 60 90 90 90 120	93 85 65 77 46 45 55 40	65 (67) 69 (70) 55 (60) 63 (67) 42 (46) 32 (42) 49 (53) 30 (35)

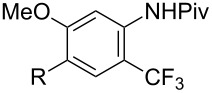	H^b^ Cl	45 100	70^b^ 67	40 (48)^b^ 40 (55)

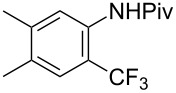		80	71	48 (57)

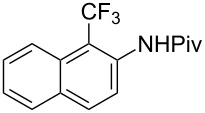		60	60	54 (58)

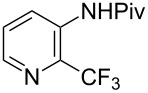		100	---	51 (---)

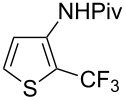		100	---	86 (---)

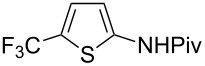		100	---	52 (---)

^a^Reaction time: 36 h. ^b^The isomer bearing CF_3_ *para* to the amide group was also produced in 16% isolated yield.

**Figure 7:**
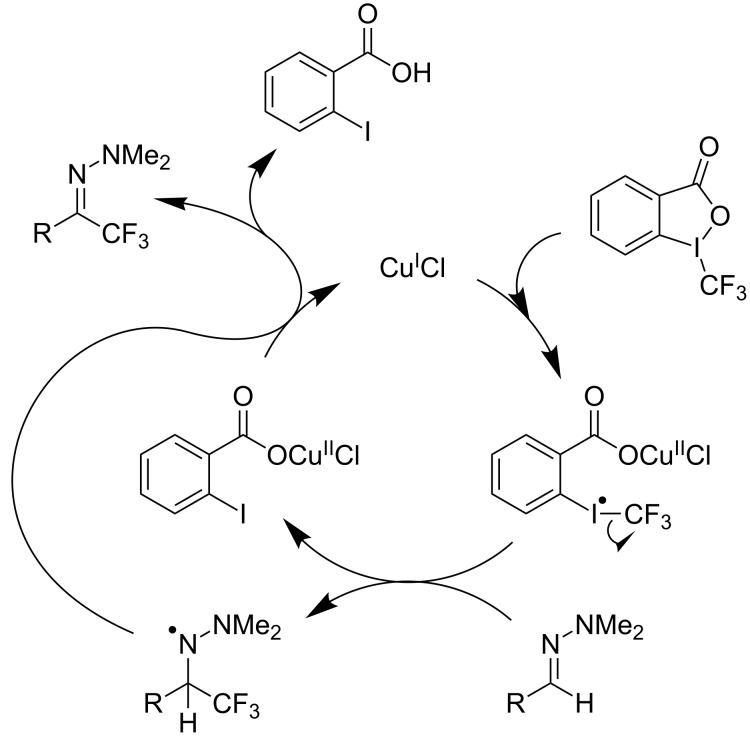
Proposed catalytic cycle for the copper-catalyzed trifluoromethylation of *N*,*N*-disubstituted (hetero)arylhydrazones by D. Bouyssi, O. Baudoin et al. [85].

As demonstrated recently by D. Bouyssi, O. Baudoin and coworkers, copper proved also able to catalyze the introduction of a CF_3_ group at the “imino” C–H bond of *N*,*N*-disubstituted (het)arylhydrazones [85]. Here again, a simple system consisting of Togni’s reagent and 10 mol % of copper(I) chloride could trifluoromethylate substrates efficiently without any additive nor heating, and in a short reaction time. The substituents on the terminal nitrogen atom had a strong influence on the reaction. Two alkyl substituents on nitrogen gave far better results than a single one; benzyl as well as phenyl groups were tolerated, although giving lower yields. A broad substitution pattern on the (hetero)aryl ring was compatible with the reaction, and the “imino” C–H was selectively trifluoromethylated (Table 18). When carrying out the reaction in the presence of TEMPO, the desired reaction was almost completely shut down, while a nearly quantitative ^19^F NMR yield was determined for the formation of the TEMPO-CF_3_ adduct, giving evidence for a radical mechanism (Figure 7).

**Table 18:** Baudoin’s Cu-catalyzed trifluoromethylation of *N*,*N*-disubstituted (het)arylhydrazones [85].

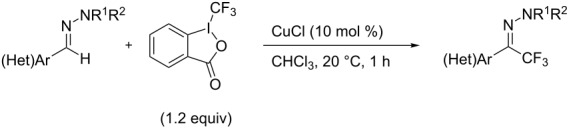				

Product		Yield (%)^a^	Product	Yield (%)^a^

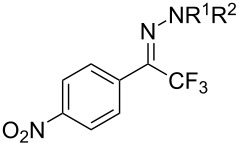	NMe_2_ NBn_2_ NPh_2_ NHMe 1-piperidinyl 4-morpholinyl	96 61 30 ---^b^ 88 86	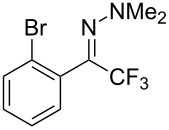	82

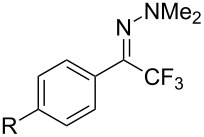	CN F OH NMe_2_	99 56^c^ 65^d^ 56	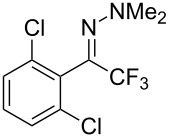	85

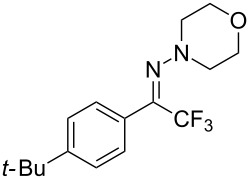		73	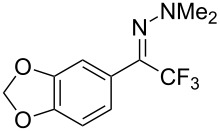	85

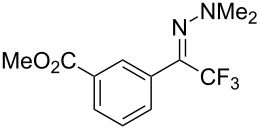		82	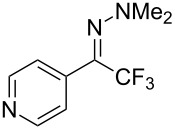	74

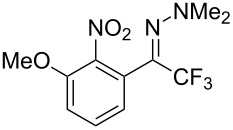		90	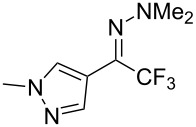	75

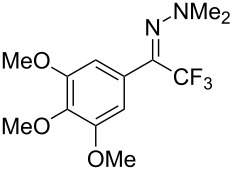		80	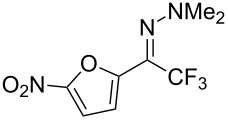	60^e^

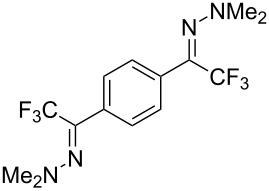		68^d^		

^a^Yields for isolated compounds. ^b^Complex crude mixture. ^c^Volatile compound (78% NMR yield). ^d^CuI was used as catalyst in DCM. ^e^18 h reaction time; additional CuCl (10 mol %) and Togni’s reagent (0.5 equiv) were added after 15 h (68% conversion) to complete the reaction.

**Figure 8:**
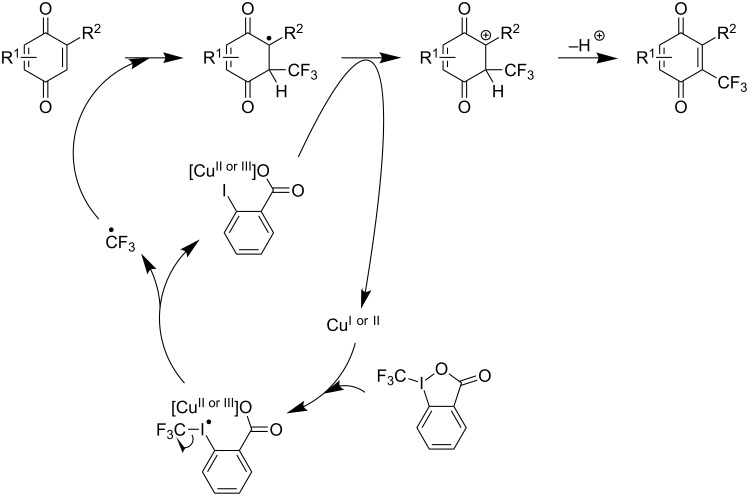
Proposed catalytic cycle by Y. Zhang and J. Wang et al. for the copper-catalyzed trifluoromethylation of quinones [87].

Very recently, K. J. Szabó et al. [86] and Y. Zhang and J. Wang et al. [87] simultaneously published their work on the trifluoromethylation of variously functionalized quinones. Both groups observed the inefficiency of Umemoto’s sulfonium reagents in this reaction, whereas Togni’s benziodoxolone reagent gave the best results. Y. Zhang, J. Wang and coworkers used 20 mol % of copper(I) iodide in a 1:1 *t-*BuOH/DCM solvent system at 55 °C with 2 equivalents of Togni’s reagent [87]. On the other hand, K. J Szabó et al. had to use stoichiometric amounts of copper(I) cyanide and catalytic bis(pinacolato)diboron to achieve optimal yields, but a catalytic amount of CuCN could also produce the desired trifluoromethylated products if stoichiometric potassium or tetrabutylammonium cyanide were also added to the reaction medium [86]. Both groups noticed that in the presence of TEMPO as radical scavenger, the reaction was seriously inhibited, and TEMPO-CF_3_ was obtained in high yields. Y. Zhang and J. Wang et al. proposed a plausible mechanism to account for this observation [87]. The mechanism is related to those described above for pivanilides (C. Chen, C. Xi et al.) or hydrazones (D. Bouyssi, O. Baudoin et al.) (Figure 8).

**Figure 9:**
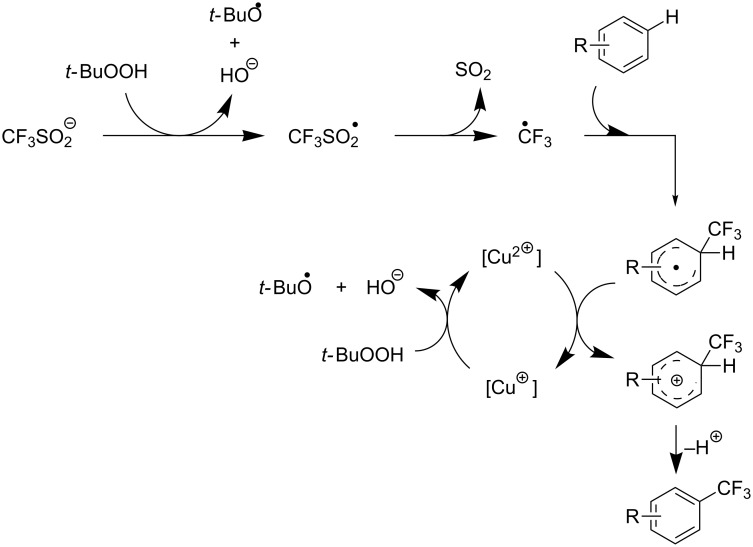
Mechanistic rationale for the trifluoromethylation of arenes in presence of Langlois’s reagent and a copper catalyst (B. R. Langlois et al.) [88].

**3.2.3 Perfluoroalkylation of Csp^2^–H bonds by means of a CF_3_-radical source.** Clearly Togni’s electrophilic reagent is able to generate the CF_3_^•^ radical in the presence of catalytic copper(I) sources. However, generation of this radical and its use in copper-catalyzed trifluoromethylation of sp^2^-C–H bonds was described much earlier by B. R. Langlois et al. [88]. In their report, *N*-acetylpyrrole and a series of electron-rich benzenes were functionalized in moderate yields by using sodium trifluoromethanesulfinate (Langlois’s reagent) and *tert*-butyl peroxide with 10 mol % of copper(II) triflate (Table 19). The supposed mechanism implies single electron transfers where *t-*BuOOH and Cu(OTf)_2_ serve as oxidants (Figure 9).

**Table 19:** Cu-catalyzed trifluoromethylation with Langlois’s sodium trifluoromethanesulfinate as CF_3_ radical source [88].

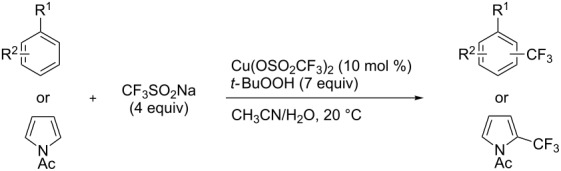			

Product	CH_3_CN/H_2_O ratio	Isolated Yield (%)	Product ratio

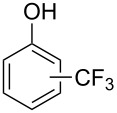	1:0	45	*o*/*m*/*p* = 4:1:6
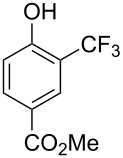	1:0	21	---
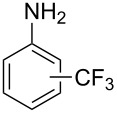	1:2	13	n.p. (2 isomers)
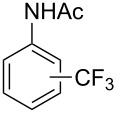	1:2	52	*o*/*m*/*p* = 4:1:2
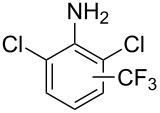	1:0	29	4-CF_3_/3-CF_3_ = 3:1
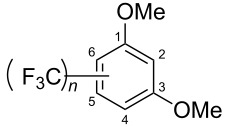	1:0	90^a^	2-CF_3_/6-CF_3_/2,6-(-CF_3_)_2_/4,6-(-CF_3_)_2_ = 23:58:4:2.5
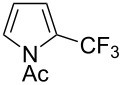	n.p.	35	---

^a^Reaction carried out under N_2_. n.p. = not precized by the authors.

**Scheme 7:**
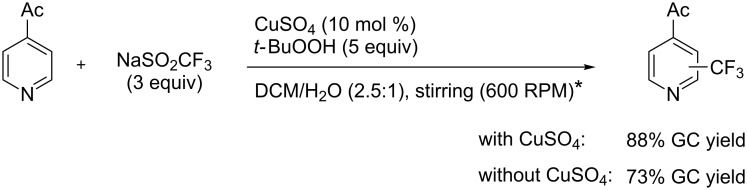
Trifluoromethylation of 4-acetylpyridine with Langlois’s reagent by P. S. Baran et al. (* Stirring had a strong influence on the reaction efficiency; see the original article for details) [89].

Interestingly, Langlois’s reagent was also used recently by P. S. Baran et al. for the generation of the CF_3_^•^ radical and trifluoromethylation of heteroaromatic compounds [89]. Although copper(II) sulfate (10 mol %) led to improved yields, trifluoromethylation was found to proceed in the absence of added metallic catalysts, and it is believed that traces only of metals present in the CF_3_ source are sufficient to initiate the reaction (Scheme 7).

**Scheme 8:**
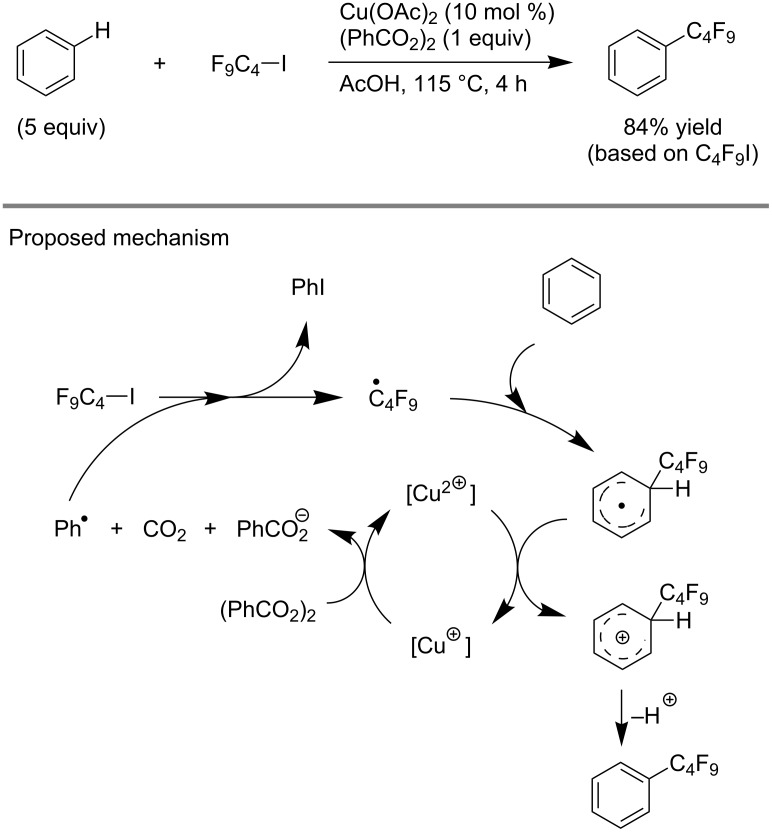
Catalytic copper-facilitated perfluorobutylation of benzene with C_4_F_9_I and benzoyl peroxide [90].

Finally, F. Minisci et al. showed that catalytic amounts of Cu(II) salts could improve the yields in the perfluoroalkylation of arenes by perfluoroalkyl iodides in the presence of benzoyl peroxide (Scheme 8). The copper salts are believed to speed up the process by superimposing a redox chain to the radical chain [90].

**Figure 10:**
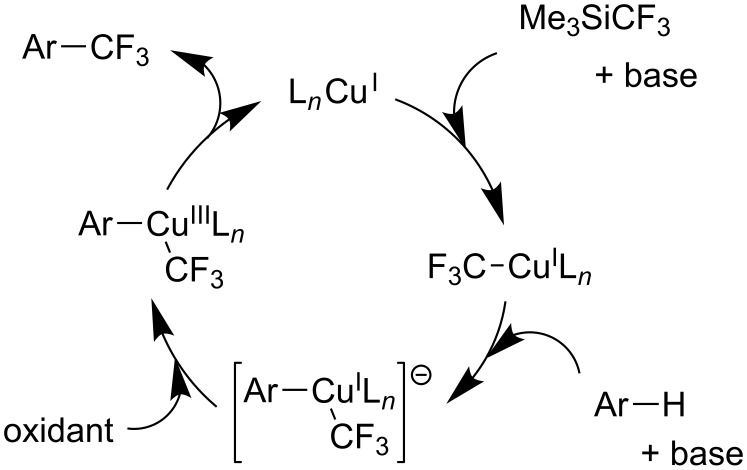
F.-L. Qing et al.’s proposed mechanism for the copper-catalyzed trifluoromethylation of (hetero)arenes with the Ruppert–Prakash reagent [91].

**3.2.4 Trifluoromethylation of Csp^2^–H bonds by means of a nucleophilic CF_3_-source.** To the best of our knowledge, there is only one report in the literature by L. Chu and F.-L. Qing, where catalytic copper was used in the trifluoromethylation of sp^2^-C–H bonds by a nucleophilic CF_3_-releasing reagent [91]. In this paper, heteroarenes or arenes bearing acidic sp^2^-C–H bonds were trifluoromethylated by the Ruppert–Prakash reagent in presence of catalytic copper(II), a base and an oxidant. The reaction conditions had to be slightly customized for each class of substrates. The methodology was first developed for 2-substituted 1,3,4-oxadiazoles (Cu(OAc)_2_/1,10-phenanthroline/*t-*BuONa/NaOAc/air, Table 20), then extended to benzo[*d*]oxazoles, benzo[*d*]imidazoles, benzo[*d*]thiazoles, imidazoles and polyfluorobenzenes (same system but di-*tert*-butyl peroxide as oxidant instead of air, Table 21); the nature of the copper(II) salt, the base and the oxidant had to be reassessed for the reaction of indoles (Cu(OH)_2_/1,10-phenanthroline/KF/Ag_2_CO_3_). Interestingly, the results obtained for indoles could be directly compared to those reported by G. Liu and coworkers for the analogous, Pd-catalyzed, TMSCF_3_-induced trifluoromethylation of the same substrates (section 3.1.4). It appears that the Cu-based system gave generally higher yields. L. Chu and F.-L. Qing compared stoichiometric and catalytic experiments and came to the conclusion that the reaction most probably proceeded via a trifluoromethylcopper(I) species, which would activate the C–H bond of the substrate and then be oxidized to a copper(III) complex, finally releasing the trifluoromethylated product by reductive elimination (Figure 10).

**Table 20:** Qing’s Cu-catalyzed trifluoromethylation of 1,3,4-oxadiazoles with the Ruppert–Prakash reagent [91].

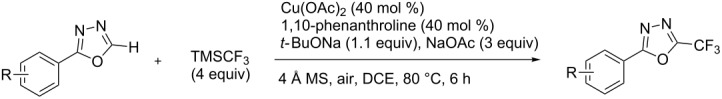		

Product		Isolated Yield (%)

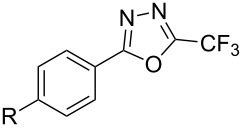	H Me *t-*Bu OMe CF_3_ NO_2_ CO_2_Me Cl	89 83 91 87 72 43 81 83
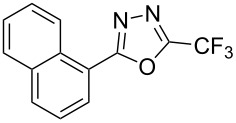		85

**Table 21:** Extension of Qing’s Cu-catalyzed trifluoromethylation to benzo[*d*]oxazoles, benzo[*d*]imidazoles, benzo[*d*]thiazoles, imidazoles and polyfluorobenzenes [91].

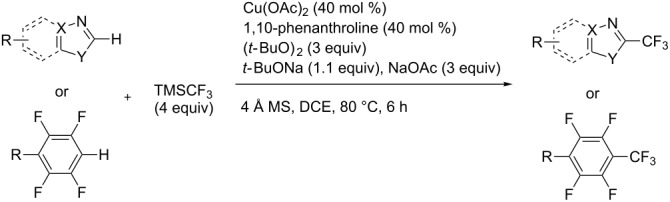					

Product		Yield (%)^a^	Product		Yield (%)^a^

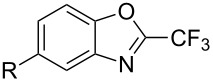	Me Ph Br Cl	72 88 (95^b^) 58 75	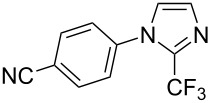		30^b^

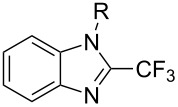	Me (CH_2_)_2_CH=CH_2_	57^b^ 32^b^	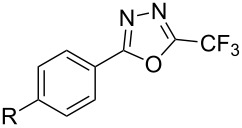	H OMe CF_3_	81 83 69

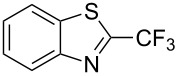		74^b^	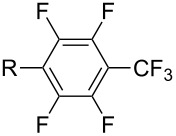	F 4-MeO-C_6_H_4_	93^c^ 63^b^

^a^Isolated yields, unless otherwise noted. ^b^Some starting material was also recovered. ^c 19^F NMR yield using an internal standard.

**3.2.5 Trifluoromethylation of arylboron reagents with a nucleophilic CF_3_-source under oxidative conditions.** F.-L. Qing reported on the first Cu-catalyzed cross-coupling of aryl- and alkenylboronic acids with TMSCF_3_ under oxidative conditions (Table 22) [34,92]. Although the detailed mechanism remains to be elucidated, the authors presume that the reaction proceeds via generation of CuCF_3_ followed by transmetallation with the arylboronic acid. The diamine stabilizes the CuCF_3_ species. This facilitates the oxidation to Cu(II) or Cu(III) species which undergo facile reductive elimination.

**Table 22:** Cu-catalyzed cross-coupling of (hetero)aryl- and alkenylboronic acids with TMSCF_3_ under oxidative conditions [92].

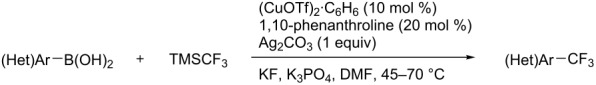			

Compound	Yield (%)	Compound	Yield (%)

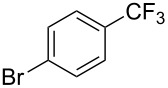	58	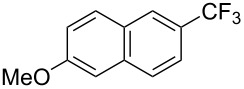	81
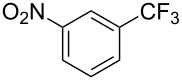	74	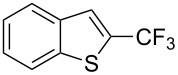	65
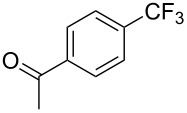	78	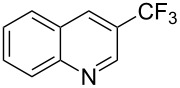	49
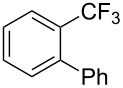	72	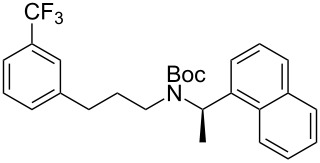	56

**3.2.6 Trifluoromethylation of arylboron reagents with an electrophilic CF_3_-source.** L. Liu found that the copper-catalyzed trifluoromethylation of aryl, heteroaryl, and vinylboronic acids with Umemoto's trifluoromethyl dibenzosulfonium salt can be performed under mild conditions and with tolerance towards a variety of functional groups (Table 23) [93].

**Table 23:** Cu-catalyzed trifluoromethylation of aryl, heteroaryl, and vinyl boronic acids with Umemoto's trifluoromethyl dibenzosulfonium salt [93].

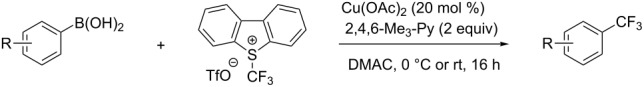					

Compound	Yield (%)	Compound	Yield (%)	Compound	Yield (%)

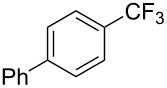	70	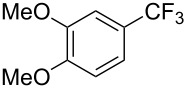	39	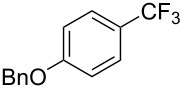	65
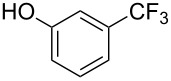	60	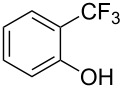	30	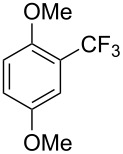	65
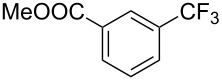	57	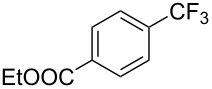	52	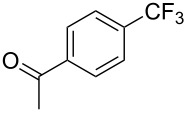	57
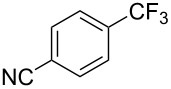	70	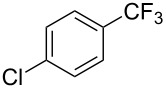	78	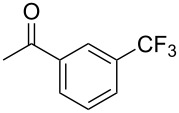	50
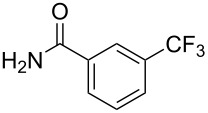	40	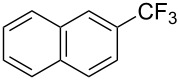	59	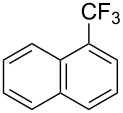	62
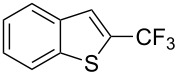	64	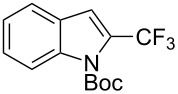	54	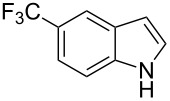	51
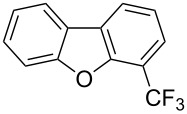	65	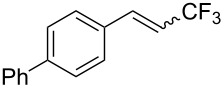	46		

Q. Shen reported on the copper-catalyzed trifluoromethylation of aryl- and alkenylboronic acids employing Togni's hypervalent iodine reagent. The reaction proceeds in good to excellent yields affording a wide range of trifluoromethylated products (Table 24) [94].

**Table 24:** Cu-catalyzed trifluoromethylation of aryl- and alkenylboronic acids employing Togni's hypervalent iodine reagent [94].

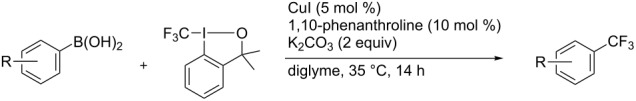					

Compound	Yield (%)	Compound	Yield (%)	Compound	Yield (%)

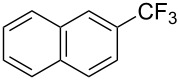	80	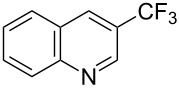	53	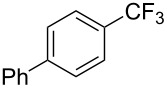	90
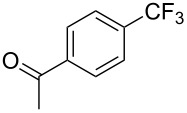	85	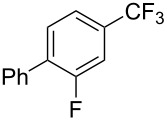	90	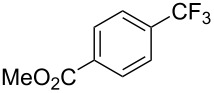	90
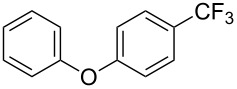	90	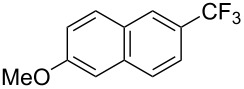	95	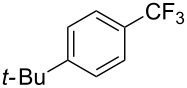	90
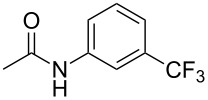	70	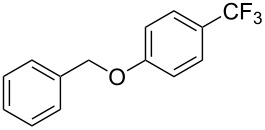	85	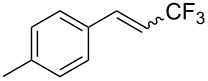	50
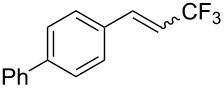	75	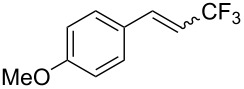	55	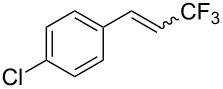	70
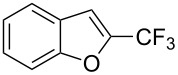	76	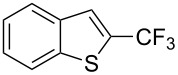	73	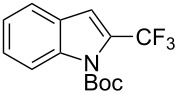	80

A similar approach has been reported by K.-W. Huang and Z. Weng employing organotrifluoroborates under base free conditions (Table 25) [95].

**Table 25:** Cu-catalyzed trifluoromethylation of organotrifluoroborates with Togni's hypervalent iodine reagent [95].

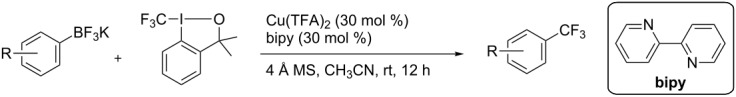					

Compound	Yield (%)	Compound	Yield (%)	Compound	Yield (%)

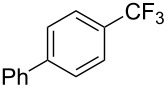	95	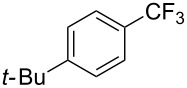	91	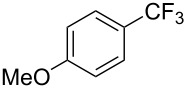	60
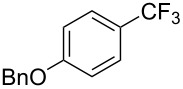	92	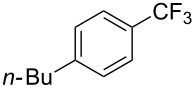	89	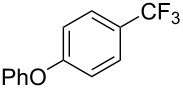	94
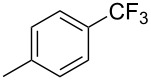	69	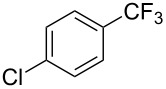	50	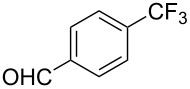	39
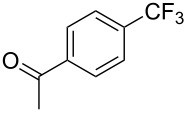	42	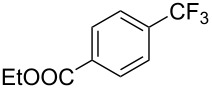	72	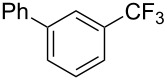	82
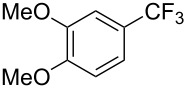	65	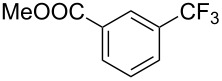	81	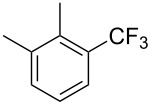	65
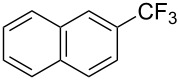	51	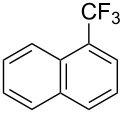	50	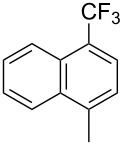	70
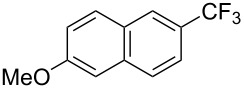	65				

**3.2.7 Radical trifluoromethylation of arylboron reagents.** In contrast to previous approaches where relatively expensive trifluoromethylsilanes are required such as Ruppert–Prakash reagent (TMSCF_3_) or TESCF_3_ to generate a CF_3_-nucleophile, and *S*-(trifluoromethyl)thiophenium salts or Togni’s reagent to generate a CF_3_^+^-electrophile, an alternative approach has recently been reported, by different groups, where highly reactive CF_3_ radicals are generated.

**Figure 11:**
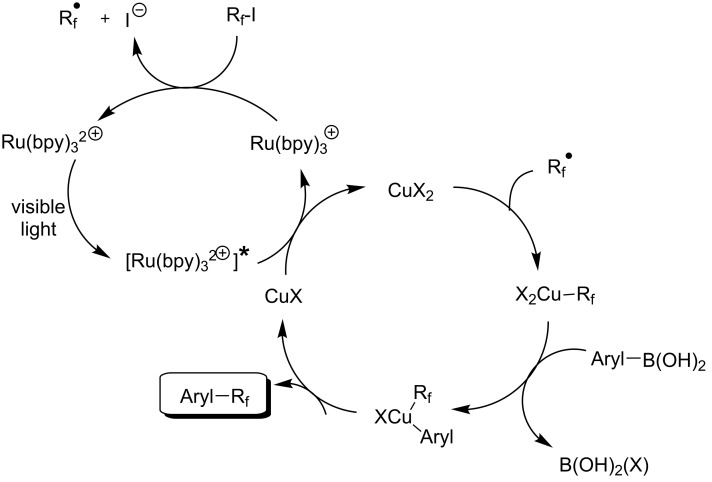
Mechanism of the Cu-catalyzed/Ru-photocatalyzed trifluoromethylation and perfluoroalkylation of arylboronic acids [96].

M. S. Sanford has developed a mild and general approach for the Cu-catalyzed/Ru-photocatalyzed trifluoromethylation and perfluoroalkylation of arylboronic acids [96]. The ruthenium-bipyridyl complex plays a double role in this reaction, namely the generation of the CF_3_ radical, and the oxidation of Cu(I) to Cu(II) under photoexcitation. Both products then combine to afford a Cu(III)CF_3_ species, which undergoes transmetallation with the arylboronic acid. Finally, reductive elimination from Cu(III)(aryl)(CF_3_) affords the desired aryl-CF_3_ product (Figure 11 and Table 26).

**Table 26:** Sanford’s Cu-catalyzed/Ru-photocatalyzed trifluoromethylation and perfluoroalkylation of (hetero)arylboronic acids [96].

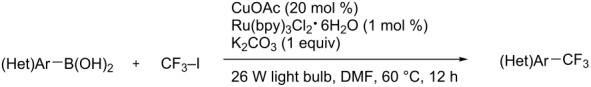					

Compound	Yield (%)	Compound	Yield (%)	Compound	Yield (%)

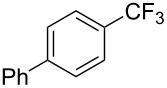	70	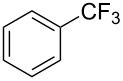	70	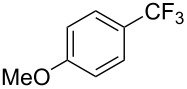	84
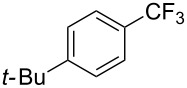	72	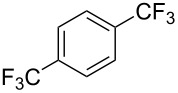	64	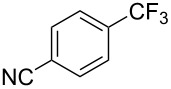	65
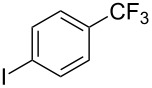	64	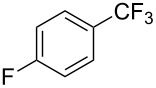	93	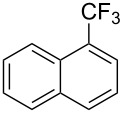	42
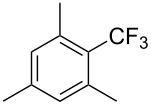	39	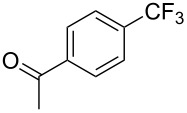	64	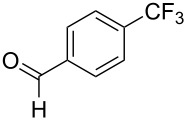	63
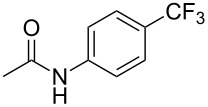	68	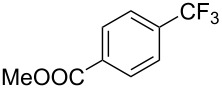	68	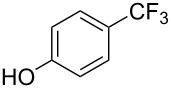	64
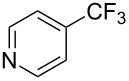	64	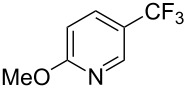	66	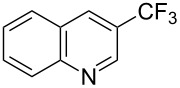	67
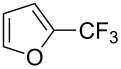	48	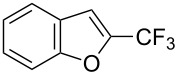	56	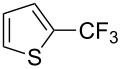	54
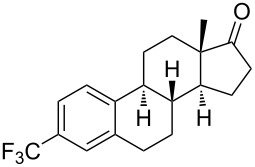	80				

**Figure 12:**
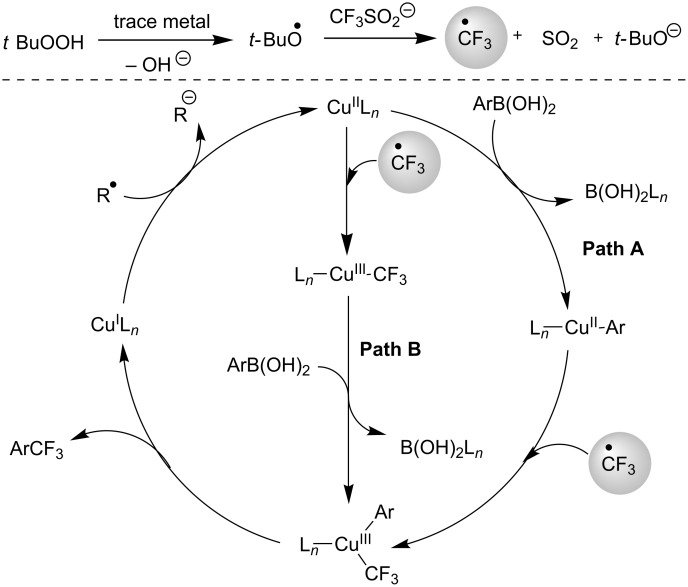
Proposed mechanism for the Cu-catalyzed trifluoromethylation of aryl- and vinyl boronic acids with NaSO_2_CF_3_ [97].

M. Beller et al. investigated the copper-catalyzed trifluoromethylation of aryl and vinyl boronic acids with in situ generated CF_3_-radicals using NaSO_2_CF_3_ (Table 27 and Table 28) [97]. The CF_3_ radical is generated from the reaction of TBHP (*t-*BuOOH) with NaSO_2_CF_3_. Transmetallation of the arylboronic acid with the Cu(II) species gives an aryl copper(II) complex. Combination of the CF_3_ radical with this complex affords the arylcopper(III)CF_3_ intermediate (Figure 12, Path A). Reductive elimination then gives the trifluoromethylated product and a Cu(I) complex which is re-oxidized to the active Cu(II) catalyst. The authors postulate also a second mechanism in which CF_3_ radicals react with the Cu(II) catalyst to give the aryl copper(III) complex. This is followed by transmetallation with the aryl- or vinylboronic acid affording the same intermediate proposed in Path A (Figure 12, Path B).

**Table 27:** Cu-catalyzed trifluoromethylation of (hetero)arylboronic acids [97].

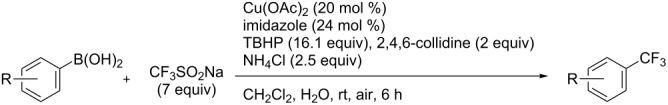					

Compound	Yield (%)	Compound	Yield (%)	Compound	Yield (%)

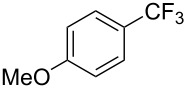	74	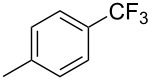	66	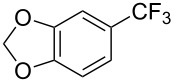	61
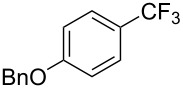	73	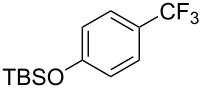	69	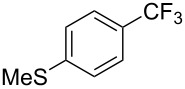	47
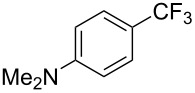	39	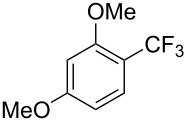	68	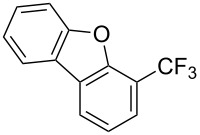	53
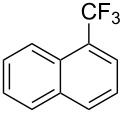	60	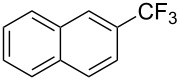	57	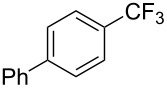	58
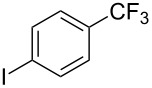	58	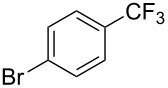	41	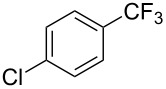	39
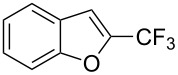	63	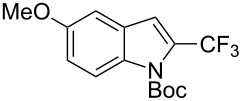	34		

**Table 28:** Cu-catalyzed trifluoromethylation of vinylboronic acids [97].

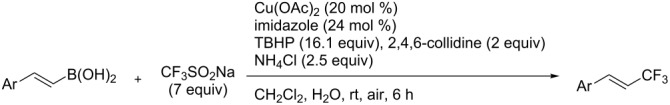					

Compound	Yield (%)	Compound	Yield (%)	Compound	Yield (%)

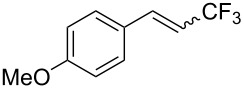	60	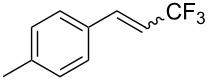	65	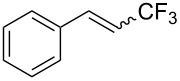	67
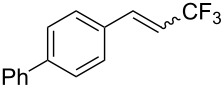	56	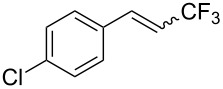	70	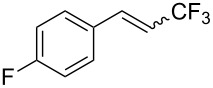	70
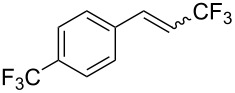	66				

**3.2.8 Trifluoromethylation of α,β-unsaturated carboxylic acids.** Carboxylic acids have often been reported as convenient reactants for metal-catalyzed decarboxylative cross-coupling reactions. The methodology developed by J. Hu et al. for the difluoromethylation of α,β-unsaturated carboxylic acids (section 2.1) has also been applied for the introduction of a CF_3_ moiety [61]. Togni’s reagent was used as the electrophilic source of CF_3_ and reacted with 4 equivalents of the (*E*)-vinylcarboxylic acid in the presence of a Lewis acid catalyst (CuF_2_·2H_2_O). Moderate to good yields were obtained for the transformation, but a slight erosion of the configuration of the double bond was observed in some cases (Table 29). The choice of the electrophilic trifluoromethylating agent seems to be crucial as no reaction was observed with Umemoto’s reagent.

**Table 29:** Cu-catalyzed C–CF_3_ bond formation on α,β-unsaturated carboxylic acids through decarboxylative fluoroalkylation [61].

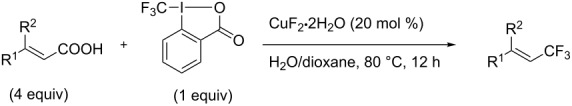					

Compound	Yield (%)	Compound	Yield (%)	Compound	Yield (%)

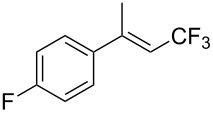	42	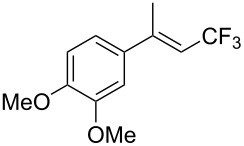	74	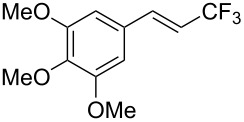	66
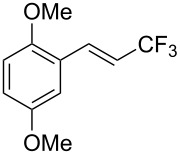	60	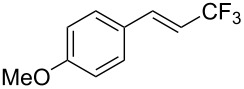	70	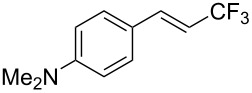	60
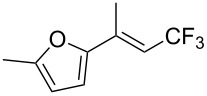	62	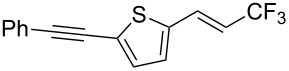	52	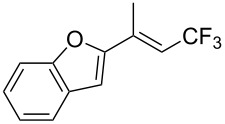	44
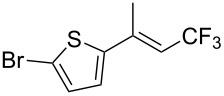	60	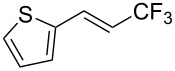	52		

Recently, Z.-Q. Liu et al. reported on a direct formation of C–CF_3_ bonds by using Langlois’s reagent as a stable and inexpensive electrophilic trifluoromethyl radical source to access trifluoromethyl-substituted alkenes [62]. Cinnamic acids were reacted with sodium trifluoromethanesulfinate and a catalytic amount of copper(II) sulfate in the presence of *tert-*butyl hydroperoxide (TBHP) as the radical initiator. The reaction was achieved with α,β-unsaturated carboxylic acids bearing electron-donating groups, as well as with heteroarene substituted acrylic acids, and the desired products were isolated in modest to good yields (Table 30). Steric effects do not appear to have an influence on the outcome of the reaction.

**Table 30:** Cu-catalyzed decarboxylative trifluoromethylation of α,β-unsaturated carboxylic acids with sodium trifluoromethanesulfinate [62].

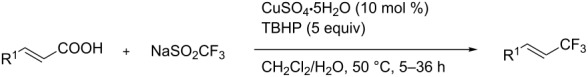					

Compound	Yield (%)	Compound	Yield (%)	Compound	Yield (%)

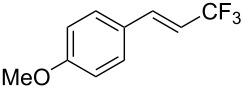	80	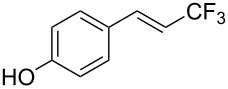	78	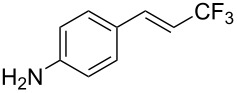	59
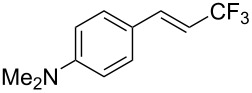	79	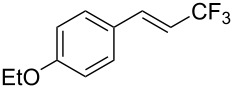	60	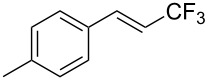	56
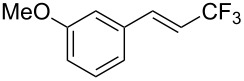	52	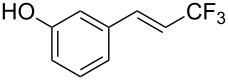	64	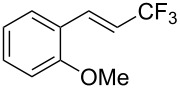	65
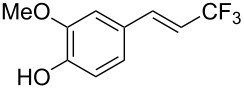	82	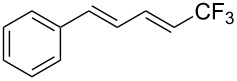	48	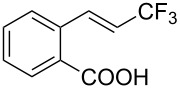	68
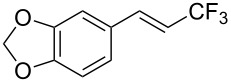	72	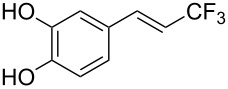	78	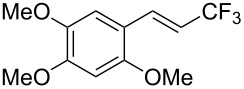	80
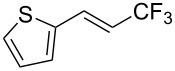	42	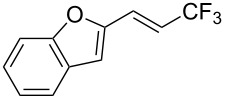	46	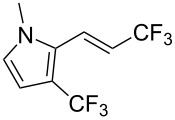	42

**Figure 13:**
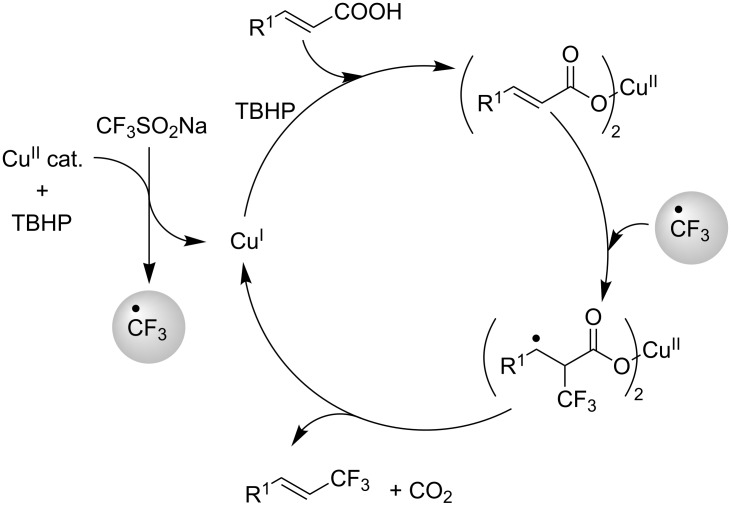
Possible mechanism for the Cu-catalyzed decarboxylative trifluoromethylation of cinnamic acids [62].

The radical CF_3_^•^ is generated by the reaction of TBHP with NaSO_2_CF_3_ and the catalytic source of Cu(II). The Cu(I) reduced from the former step reacts with the cinnamic acid in the presence of TBHP to afford a cupric cinnamate, which then undergoes the addition of the trifluoromethyl radical to the double bond. The CF_3_-substituted alkene is finally obtained after elimination of carbon dioxide and Cu(I) (Figure 13).

#### 3.3 Catalysis by other metals than Pd and Cu

**Scheme 9:**
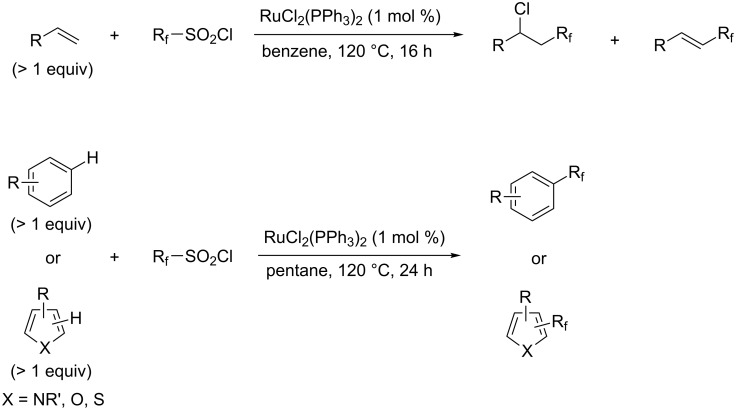
Ruthenium-catalyzed perfluoroalkylation of alkenes and (hetero)arenes with perfluoroalkylsulfonyl chlorides (N. Kamigata et al.) (R_f_ = CF_3_, C_6_F_13_) [101].

**3.3.1 Ru-catalyzed perfluoroalkylation of Csp^2^–H bonds.** More than two decades ago, the group of N. Kamigata pursued extensive investigations on the perfluoroalkylation of alkenes, aromatics and heteroaromatics catalyzed by Ru(II)Cl_2_(PPh_3_)_3_ [98–104]. In the course of their initial studies [98,100] aimed at the perfluoroalkylchlorination of terminal alkenes, they noticed that the corresponding 1-perfluoroalkyl-subsituted alkenes were sometimes obtained along with the desired addition products (Scheme 9).

**Figure 14:**
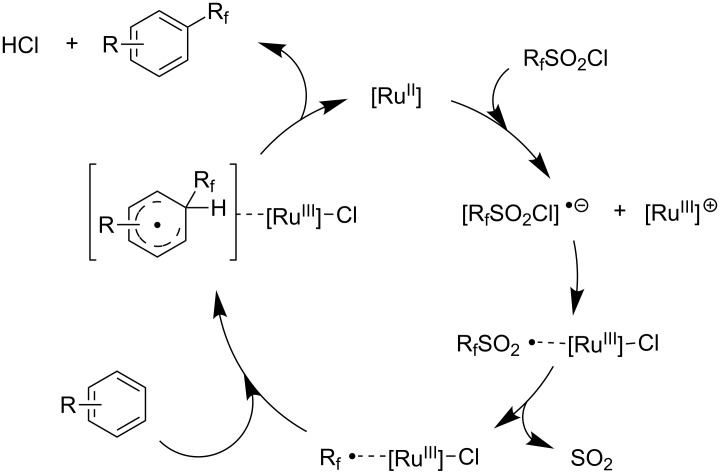
N. Kamigata et al.’s proposed mechanism for the Ru-catalyzed perfluoroalkylation of alkenes and (hetero)arenes with perfluoroalkylsulfonyl chlorides [100].

Afterwards, N. Kamigata et al. applied this system to arenes [99] and heteroarenes (furans, pyrroles and thiophenes) [102–104] and gave a full account of this work (Scheme 9) [101]. Monosubstituted benzenes gave mixtures of the *ortho*-, *meta*- and *para*-isomers. The reaction was much more regioselective in the case of thiophenes, where 2-perfluoroalkylated products were obtained, as long as at least one of the positions α to sulfur was unsubstituted; otherwise β-functionalization occurred. The same comment is applicable to pyrroles bearing a small group on nitrogen, which gave the 2-perfluoroalkylated compound as the major product. For instance, *N*-TMS-pyrrole afforded a global yield of 78% of the 2-functionalized product as a mixture of the silylated and hydrolized compounds. On the other hand, the reaction of *N*-triisopropylsilylpyrrole favoured the 3-perfluoroalkylated product over its 2-isomer, due to the steric bulk of the TIPS group. Considering the mechanism of these reactions, the authors propose a radical pathway, and more specifically a pathway where the radicals “lie in the coordination sphere of the metal”. Indeed, the present radicals led to less side-reactions – in particular, oligomerization in the case of alkenes as substrates –, which shows that they exhibit “restricted reactivity” in comparison with “that of free radicals initiated by peroxides or diazo compounds and by photoirradiation” (Figure 14) [100].

**Figure 15:**
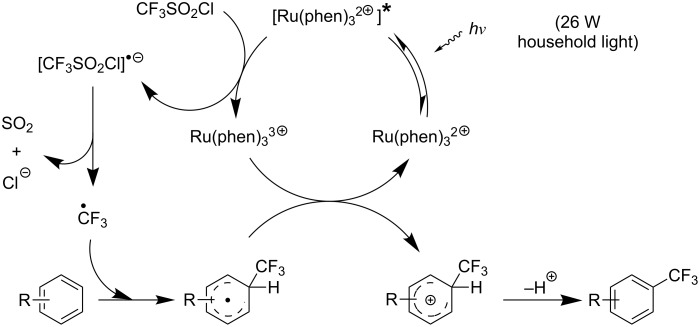
Proposed mechanism for the Ru-catalyzed photoredox trifluoromethylation of (hetero)arenes with trifluoromethanesulfonyl chloride [105].

**Figure 16:**
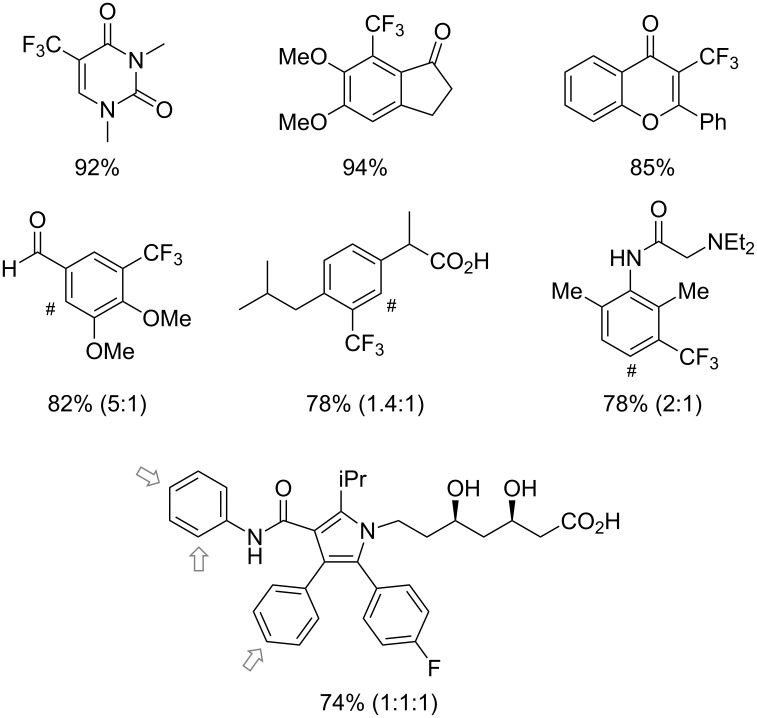
Late-stage trifluoromethylation of pharmaceutically relevant molecules with trifluoromethanesulfonyl chloride by photoredox Ru-catalysis (D. W. C. MacMillan et al.) (The position of the CF_3_ group in the other isomers produced is marked with # or an arrow) [105].

Much later, another Ru-catalysis-based methodology for the introduction of CF_3_ groups at C–H positions of arenes and heteroarenes was developed by D. W. C. MacMillan [105]. Again, trifluoromethanesulfonyl chloride was used as the CF_3_ radical source. The difference with the work of N. Kamigata et al. is that the reaction takes place under photoredox catalysis, allowing much milder reaction conditions (23 °C for D. W. C. MacMillan et al. vs 120 °C for N. Kamigata et al.). Higher yields were obtained, especially in the case of pyrroles (2-R_f_-pyrrole: 88% yield for D. W. C. MacMillan et al. (CF_3_) vs 0% for N. Kamigata et al. (C_6_F_13_); 2-R_f_-*N*-Me-pyrrole: 94% yield (CF_3_) vs 18% (C_6_F_13_)). A wide range of substrates was functionalized (Table 31). Interestingly, the late-stage trifluoromethylation of pharmaceutically relevant molecules was also carried out and proved successful (Figure 16). The mechanism of the reaction was similar to that proposed by N. Kamigata et al. (Figure 15).

**Table 31:** Ru-catalyzed photoredox trifluoromethylation of (hetero)arenes with trifluoromethanesulfonyl chloride [105].

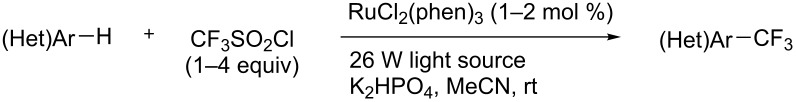					

Product^a^		Yield (%)^b^ (isomer ratio)	Product^a^		Yield (%)^b^ (isomer ratio)

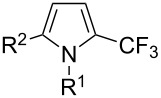	R^1^,R^2^ = H R^1^,R^2^ = Me,H R^1^,R^2^ = Boc,H R^1^,R^2^ = H,CF_3_	88 94 78 91	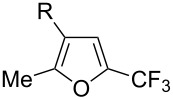	H Me	87 80

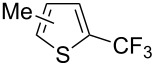	5-Me 3-Me	82 76 (3:1)^c^	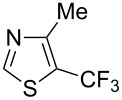		70

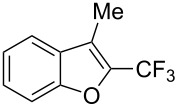		84	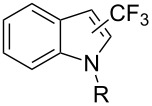	R = H; 2-CF_3_ R = Ac; 3-CF_3_	72 (4:1)^d^ 81 (3:1)^e^

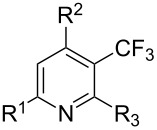	R^1^,R^2^,R^3^ = Me,H,Me R^1^,R^2^,R^3^ = Me_3_ R^1^,R^2^,R^3^ = H,H,OMe R^1^,R^2^,R^3^ = H,Me,OMe	73 81 78 (3:1)^f^ 78	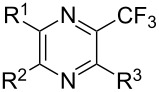	R^1^,R^2^,R^3^ = H,H,OMe R^1^,R^2^,R^3^ = Me,H,Me R^1^,R^2^,R^3^ = H,Me,Me R^1^,R^2^,R^3^ = H,Cl,Cl	82 78 94 70

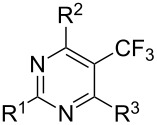	R^1^,R^2^,R^3^ = iPr,Me,OH R^1^,R^2^,R^3^ = SMe,Me,H R^1^,R^2^,R^3^ = (OMe)_3_	85 72 86	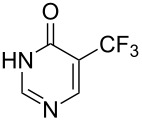		74

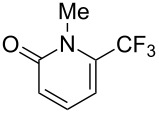		87	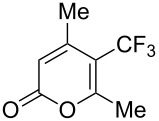		90

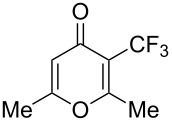		88			

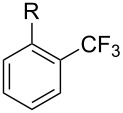	H NHBoc OMe SMe	74 80 (3:1)^g^ 84 (2:1)^g^ 73 (2:1)^g^	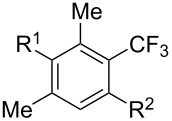	R^1^,R^2^ = H,Me R^1^,R^2^ = Br,H R^1^,R^2^ = H,H	70 75 (4:1) 77 (2:1)^h^

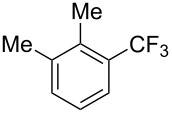		72 (2:1)	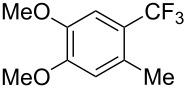		92 (5:1)^i^

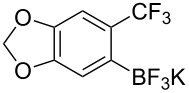		74 (2:1)^j^	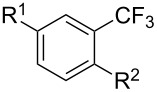	R^1^,R^2^ = Me_2_ R^1^,R^2^ = (OMe)_2_ R^1^,R^2^ = TMS,OMe R^1^,R^2^ = Me,OMe R^1^,R^2^ = *t-*Bu,Me	77 85 76 85 (4:1) 78 (5:1)

^a^The major isomer is represented. ^b^Isolated yields of the mixtures of isomers, except for volatile compounds (^19^F NMR yields). ^c^Minor isomer: 3-Me-5-CF_3_-thiophene. ^d^Minor isomer: 3-CF_3_-indole. ^e^Minor isomer: *N*-acetyl-2-CF_3_-indole. ^f^Minor isomer: 2-OMe-5-CF_3_-pyridine. ^g^Minor isomer: *para*-substituted product. ^h^Minor isomer: 1,3-Me_2_-2-CF_3_-benzene. ^i^Minor isomer: 1,2-(OMe)_2_-5-Me-3-CF_3_-benzene. ^j^Minor isomer: 4,6-disubstituted isomer.

**Figure 17:**
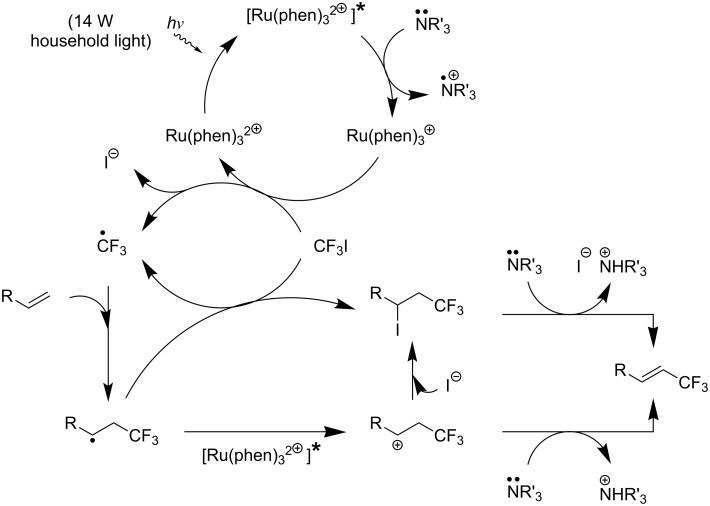
Proposed mechanism for the trifluoromethylation of alkenes with trifluoromethyl iodide under Ru-based photoredox catalysis (E. J. Cho et al.) [106].

A complementary study was published by E. J. Cho et al. in 2012 [106]. Here, terminal and internal alkene C–H bonds were trifluoromethylated under photoredox Ru-catalysis, using trifluoromethyl iodide instead of trifluoromethanesulfonyl chloride (Table 32). Interestingly, arenes were unreactive under the reaction conditions. The catalyst loading was very low (0.1 mol %) and the reactions proceeded at room temperature, giving generally high yields of the trifluoromethylalkenes. Two equivalents of DBU as an additive were optimal, since this reagent is assumed to behave both as a reductant and as a base in the proposed mechanism of the reaction. Thus, the Ru(I)/R(II) catalytic cycle is different from the mechanism proposed by D. W. C. MacMillan and coworkers (Ru(II)/Ru(III) cycle, Figure 17).

**Table 32:** Photoredox Ru-catalyzed trifluoromethylation of terminal and internal alkene C–H bonds with trifluoromethyl iodide [106].

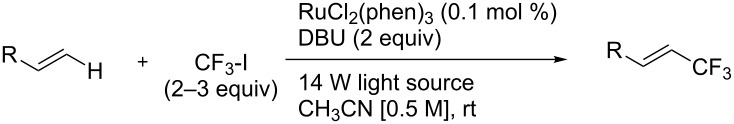				

Product		Yield (%)^a^	Product	Yield (%)^a^

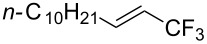		95	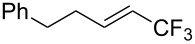	90

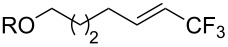	H C(O)-*n-*hept Bz C(O)NMe_2_ TBDMS Ts	80 80 93 80 89 90	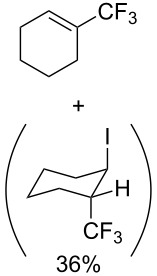	51

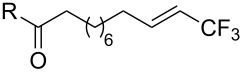	H Me	78 81	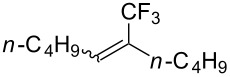	80^b^

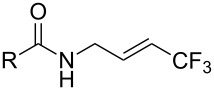	*n-*hept 4-Br-C_6_H_4_ 4-Cl-C_6_H_4_	85 83 79	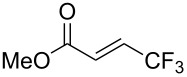	55^c^

			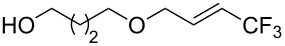	84^d^

^a^Isolated yields, unless otherwise noted. ^b^Diastereomer ratio 1.4:1. ^c 19^F NMR yield. ^d^17:1 ratio with the allyl-CF_3_ isomer.

The same group also applied this methodology to the trifluoromethylation of indoles and a couple of other heteroarenes, under closely related conditions. Trifluoromethyl iodide, catalytic Ru(II)(bpy)_3_Cl_2_ and TMEDA, as the base, were used with acetonitrile as the solvent (Table 33). Electron-deficient heteroarenes and unactivated arenes were unreactive. The mechanism is analogous to the one depicted for alkenes [106].

**Table 33:** Trifluoromethylation of indoles with trifluoromethyl iodide under Ru-based photoredox catalysis [107].

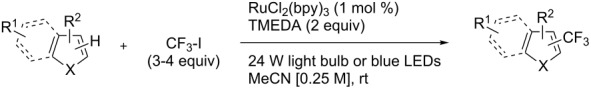			

Product	Yield (%)^a^	Product	Yield (%)^a^

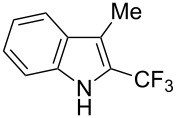	90	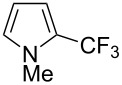	95^d^
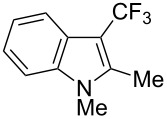	94	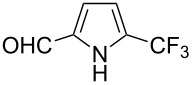	71
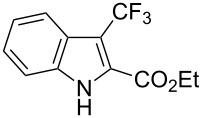	81	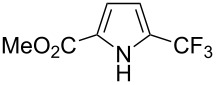	80
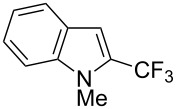	95 (1.5:1)^b^	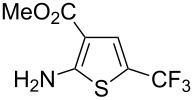	92
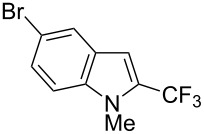	86 (1.3:1)^c^	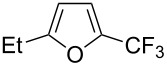	92^d^

^a^Isolated yields unless otherwise noted. ^b^As a 1.5:1 mixture with the 3-CF_3_ isomer; the major isomer is represented. ^c^As a 1.3:1 mixture with the 2-CF_3_ isomer; the major isomer is represented. ^d 19^F NMR yield.

**Scheme 10:**
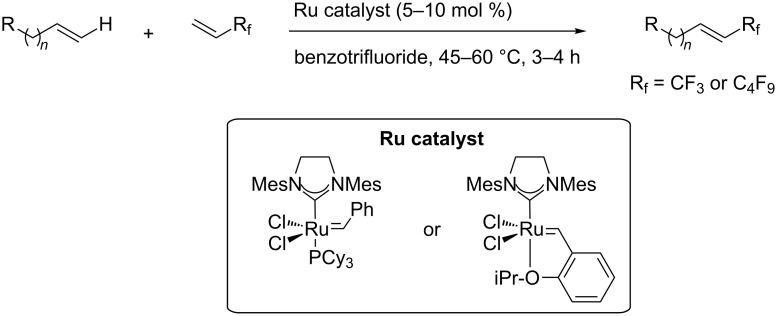
Formal perfluoroakylation of terminal alkenes by Ru-catalyzed cross-metathesis with perfluoroalkylethylenes (S. Blechert et al.) [108].

Last but not least, a completely different strategy used by S. Blechert et al. involved the cross-metathesis of terminal olefins with perfluoroalkylethylenes [108]. Thus, the reaction does not proceed through the direct introduction of C*_n_*F_2_*_n_*_+1_^+^, C*_n_*F_2_*_n_*_+1_^•^ or C*_n_*F_2_*_n_*_+1_^−^, but of a perfluoralkylmethylene (Scheme 10).

**3.3.2 Ir-catalyzed perfluoroalkylation of Csp^2^–H bonds.** As a preamble, it should be noted that D. W. C. MacMillan and E. J. Cho tested iridium complexes along with the ruthenium analogues in the photoredox catalytic reactions discussed in section 3.3.1. Although also active, the iridium catalysts showed lower selectivity and are more expensive [105–107].

**Figure 18:**
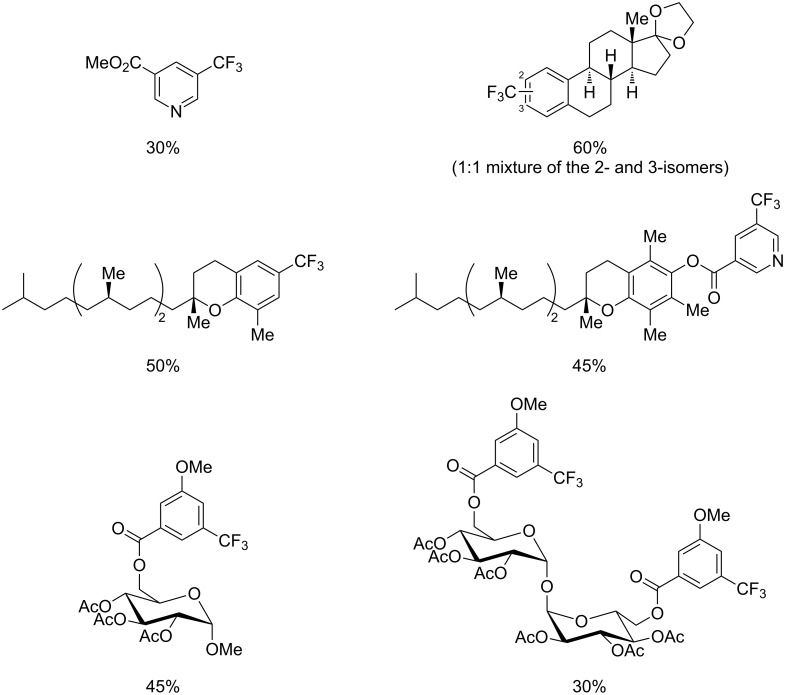
One-pot Ir-catalyzed borylation/Cu-catalyzed trifluoromethylation of complex small molecules by Q. Shen et al. [37].

A different strategy was simultaneously reported by the groups of J. F. Hartwig and Q. Shen [35,37]. The approach consists of a one-pot, two-stage reaction, with Ir-catalyzed borylation of an aromatic sp^2^-C–H bond, followed by a copper-mediated or -catalyzed perfluoroalkylation of the resulting arylboronic ester intermediate. Since the work by J. F. Hartwig et al. uses stoichiometric amounts of ex situ-prepared Cu-R_f_ reagents, we will focus on the study by Q. Shen et al. – although, once again, both are closely related. In the latter, catalytic copper(II) thiophene carboxylate was used in the second stage in the presence of 1,10-phenanthroline as a ligand; Togni’s reagent served as the CF_3_-source (Table 34). The interest of this reaction resides in the fact that the Ir-catalyzed borylation with bis(pinacolato)diboron is highly influenced by the steric bulk of the arene, and therefore leads to regioselective functionalization of the substrate. Arenes and heteroarenes, variously substituted, could undergo the reaction, including natural product related or complex small molecules (Figure 18) [37].

**Table 34:** Ir-catalyzed borylation / Cu-catalyzed perfluoroalkylation of the resulting arylboronic ester intermediate [37].

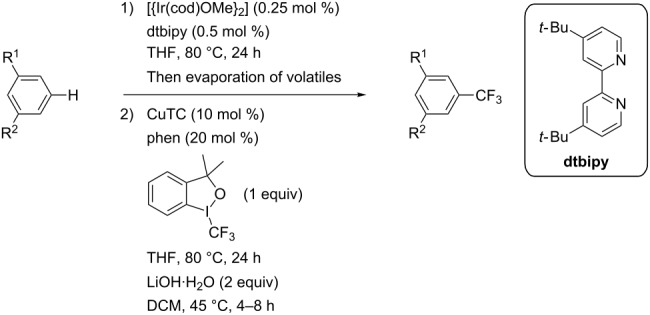					

Product		Yield (%)^a^	Product		Yield (%)^a^

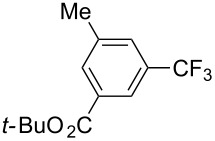	Me CF_3_ Cl	90 75 75	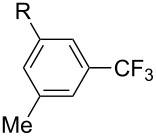	CO_2_Et OTIPS CN	80 50 70
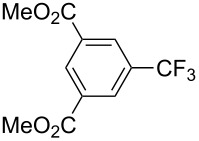		87	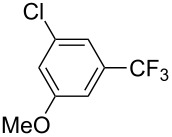		70
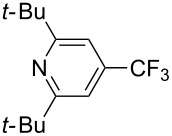		90	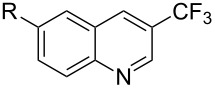	Me CO_2_-*t-*Bu	65^b^ 50
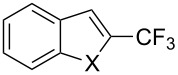	O S	72 75	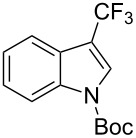		67^b^

^a^Isolated yields. ^b^1 mol % of the iridium complex and 2 mol % of the dtbipy ligand were used.

**3.3.3 Ni-catalyzed perfluoroalkylation of Csp^2^–H bonds.** Two early reports by Y.-Z. Huang et al. described Ni-catalyzed perfluoroalkylation of anilines, benzene, furan, thiophene and pyrrole using ω-chloroperfluoroalkyl iodides [109–110]. Notably, the reaction was rather selective: only *ortho*- or *para*-functionalized anilines were obtained (the ratio of which depended on the solvent), and 5-membered heterocycles all yielded the α-perfluoroalkylated products (Table 35). This selectivity differs from the one observed by N. Kamigata et al*.* in the case of ruthenium catalysts, where isomeric mixtures of α- and β-functionalized pyrroles were produced [101,104].

**Table 35:** Ni-catalyzed perfluoroalkylation of anilines, benzene, furan, thiophene and pyrrole using ω-chloroperfluoroalkyl iodides [109–110].

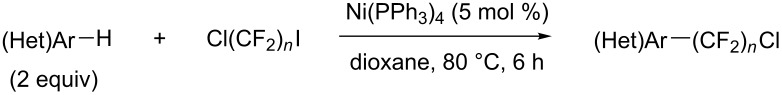				

Product	Yield (%)^a^	Product		Yield (%)^a^

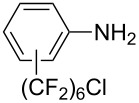	*o*-: 40 *p*-: 45	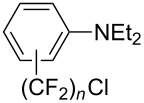	*n* = 2 *n* = 4 *n* = 6	*o*-: 22; *p*-: 65 *o*-: 21; *p*-: 63 *o*-: 16; *p*-: 50
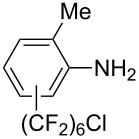	*o*-: 34 *p*-: 48	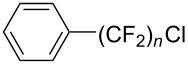	*n* = 4 *n* = 6	96^b,c,d^ 91^b,c,d^
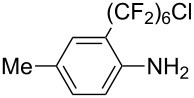	79	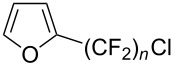	*n* = 4 *n* = 6 *n* = 8	95^b,d,e^ 93^b,d,f^ 90^b,d,g^
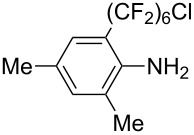	71	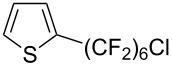		37^b,d,h^
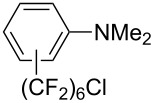	*o*-: 20 *p*-: 30	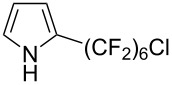		50^b,d,i^

^a 19^F NMR yield based on the perfluoroalkyl iodide. ^b^Isolated yield. ^c^Benzene itself served as solvent. ^d^NaH (2 equiv) was used as additive to trap HI. ^e^60 °C, 3 h. ^f^60 °C, 5 h. ^g^60 °C, 8 h. ^h^80 °C, 4 h. ^i^80 °C, 3 h.

**Figure 19:**
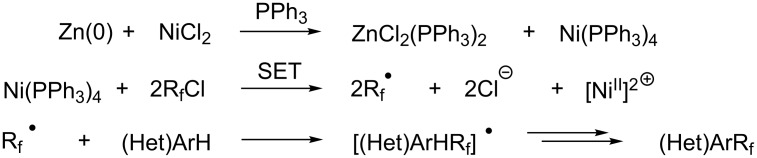
Mechanistic proposal for the Ni-catalyzed perfluoroalkylation of arenes and heteroarenes with perfluoroalkyl chlorides by Q.-Y. Chen and coworkers [111].

In 2001, Q.-Y. Chen and coworkers also reported a nickel-catalyzed methodology, with perfluoroalkyl chlorides as perfluoroalkylating reagents and in the presence of stoichiometric amounts of zinc(0) [111]. Here also, pyrrole led to a completely regioselective α-functionalization; *N*,*N*-dimethylaniline only gave the *para*-substitued product, whereas it led to a mixture of *ortho*- and *para*-perfluoroalkylated compounds with the system of Huang et al.; 4-aminoanisole yielded only the compound functionalized in the *ortho*-position with regard to the amino group (Table 36). Control experiments indicated a radical pathway for the mechanism (Figure 19).

**Table 36:** Ni-catalyzed methodology, with perfluoroalkyl chlorides as perfluoroalkylating reagents in the presence of stoichiometric zinc(0) [111].

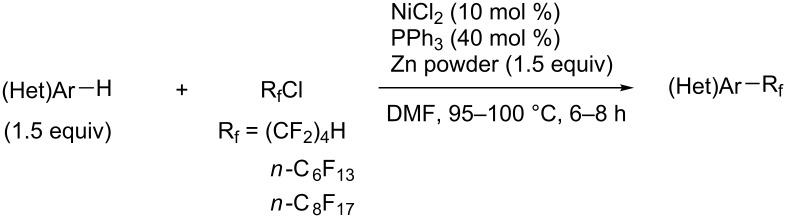			

Product	R_f_	Isolated yield (%)^a^	Isomer ratio^b^

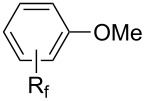	*n-*C_6_F_13_ *n-*C_8_F_17_	62 71	*o*/*m*/*p* = 44:18:38 *o*/*m*/*p* = 48:20:32

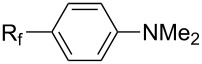	*n-*C_6_F_13_ *n-*C_8_F_17_	65 60	--- ---

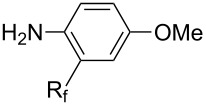	*n-*C_6_F_13_ *n-*C_8_F_17_	56 58	--- ---

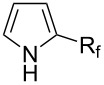	(CF_2_)_4_H *n-*C_6_F_13_ *n-*C_8_F_17_	75 78 76	--- ---

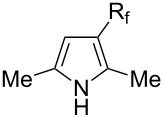	(CF_2_)_4_H *n-*C_6_F_13_ *n-*C_8_F_17_	68 70 70	--- ---

^a^Based on the starting perfluoroalkyl chloride. ^b^Determined by ^19^F NMR.

**Scheme 11:**
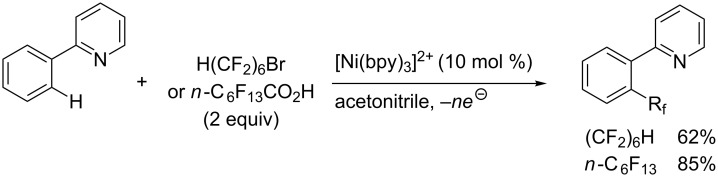
Electrochemical Ni-catalyzed perfluoroalkylation of 2-phenylpyridine (Y. H. Budnikova et al.) [71].

Finally, it is noteworthy that the electrochemical metal-catalyzed *ortho*-perfluoroalkylation of 2-phenylpyridine, which we already discussed for its Pd-catalyzed variant, is also catalyzed by nickel complexes (Scheme 11) [71]. Actually, the nickel-based systems provided higher yields than the palladium-based one (see section 3.1.3). Considering control voltamperometric experiments, a Ni(II)/Ni(III) catalytic cycle seemed to be operating.

**3.3.4 Fe-catalyzed perfluoroalkylation of Csp^2^–H bonds.** In this section, all the studies that we will discuss used substoichiometric amounts of Fenton’s reagent (FeSO_4_/H_2_O_2_) for the generation of perfluoroalkyl radicals.

Complementary work was carried out by E. Baciocchi et al. [112] and by F. Minisci et al. [90] in the perfluoroalkylation of pyrroles and indole and of benzene and anisole, respectively. The reactions were efficient (less than 30 min at room temperature). Better yields and regioselectivities were obtained for pyrrole derivatives than for benzene and anisole (Table 37 and Table 38). Interestingly, the order of preferential functionalization in the case of anisole here is *meta* ≈ *para* > *ortho*; on the contrary, all of the other perfluoroalkylation reactions of C–H bonds of anisole discussed so far and those we will discuss later [113] yielded *ortho*-perfluoroalkylated anisoles as the major products. F. Minisci and coworkers also obtained similar results when using a catalytic iron(III) salt in the presence of *tert*-butyl peroxide as oxidant.

**Table 37:** Perfluoroalkylation of pyrroles employing Fenton’s reagent [112].

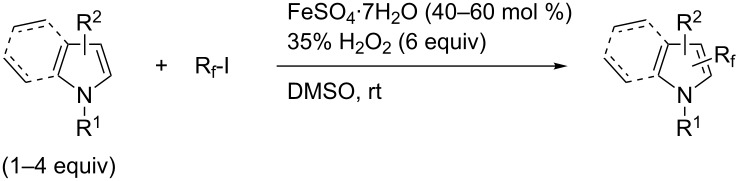					

Product	*R*_f_	Yield (%)^a^	Product	R_f_	Yield (%)^a^

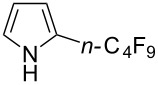	*n-*C_4_F_9_I	78^b^	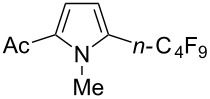	*n-*C_4_F_9_I	71
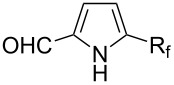	*n-*C_4_F_9_I *n-*C_3_F_7_I iC_3_F_7_I	55 64 73	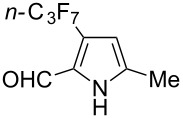	*n-*C_3_F_7_I	36
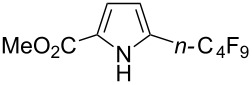	*n-*C_4_F_9_I	73	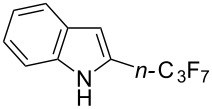	*n-*C_3_F_7_I	30

^a^Isolated yields, unless otherwise noted. ^b^GC yield.

**Table 38:** Perfluoroalkylation of benzenes or anisoles employing Fenton’s reagent [90].

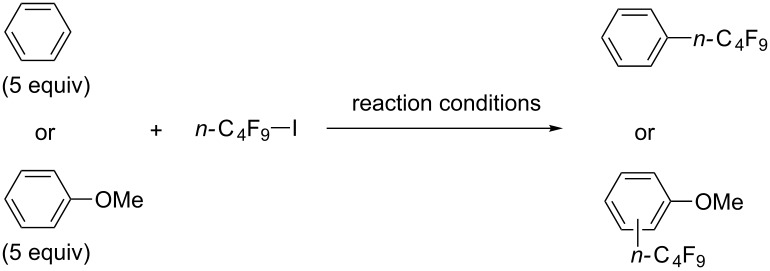				

Product	Reaction conditions	Conversion of *n-*C_4_F_9_I (%)^a^	Yield (%)^b^	Isomer ratio

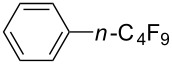	FeSO_4_•7H_2_O (70 mol %) 35% H_2_O_2_ (3 mmol) DMSO, rt	41.9	95.4	---
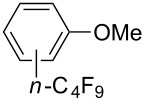		42.2	97.6	*o*/*m*/*p* = 16.1:43.4:40.5
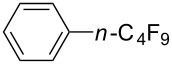	Fe(OAc)_2_OH (20 mol %) *t-*BuOOH (2 equiv) AcOH, 115 °C	58.1	96.1	---
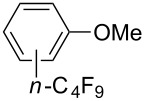		57.7	94.8	*o*/*m*/*p* = 15.5:42.8:41.7

^a^Determined by ^19^F NMR. ^b^Determined by GC or GCMS.

**Scheme 12:**
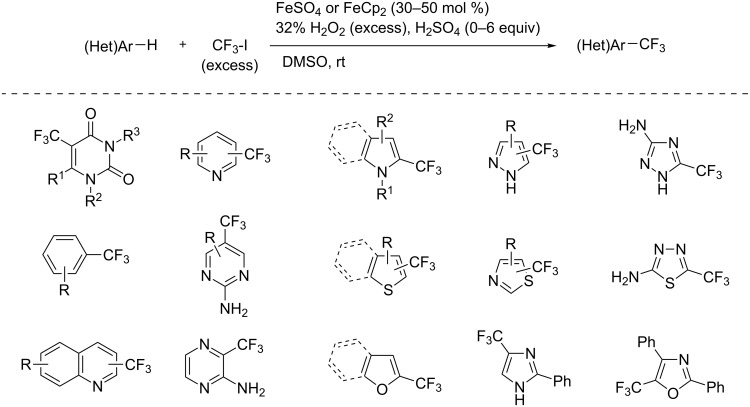
Fe(II)-catalyzed trifluoromethylation of arenes and heteroarenes with trifluoromethyl iodide (T. Yamakawa et al.) [114–115].

**Figure 20:**
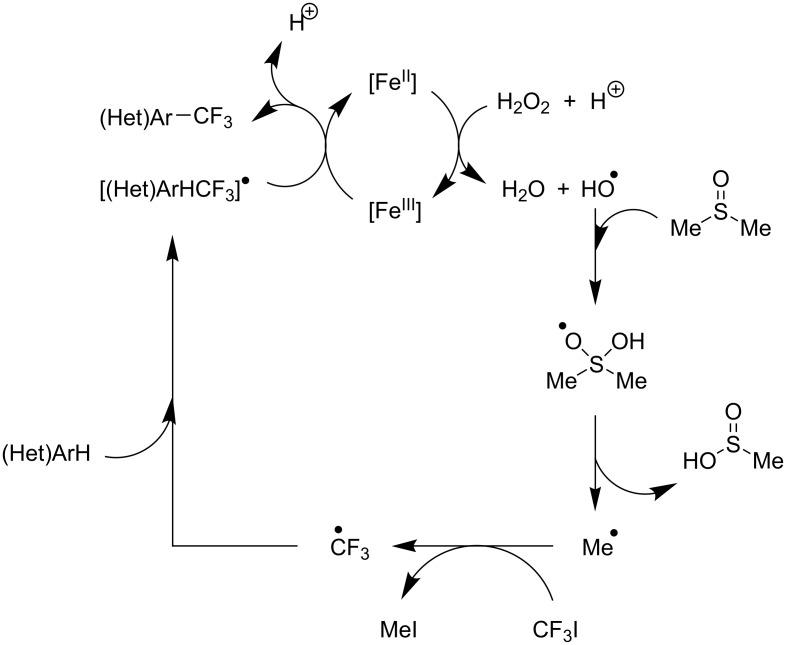
Mechanistic proposal by T. Yamakawa et al. for the Fe(II)-catalyzed trifluoromethylation of arenes and heteroarenes with trifluoromethyl iodide [114].

T. Yamakawa et al. applied this Fenton-based generation of perfluoroalkyl radicals for the trifluoromethylation of uracil derivatives [114] as well as of various arenes and heteroarenes (pyridines, pyrimidines, pyrazines, quinolines, pyrroles, thiophenes, furans, pyrazoles, imidazoles, thiazoles, oxazoles, thiadiazoles, triazoles) [115]. The yields were low to excellent, depending on the substrate (Scheme 12 and Figure 20). Iron(II) sulfate and ferrocene were used alternately as catalysts in the presence or not of sulfuric acid, but other metals proved inactive. The procedures could be adapted to larger-scale synthesis (10 g).

**3.3.5 Fe-catalyzed trifluoromethylation of arylboron reagents.** S. L. Buchwald et al. developed an iron(II)-catalyzed trifluoromethylation of potassium vinyltrifluoroborates employing Togni's reagent. The products are obtained in good yields and good to excellent *E*/*Z* ratios (Table 39) [116].

**Table 39:** Fe(II)-catalyzed trifluoromethylation of potassium vinyltrifluoroborates employing Togni's reagent [116].

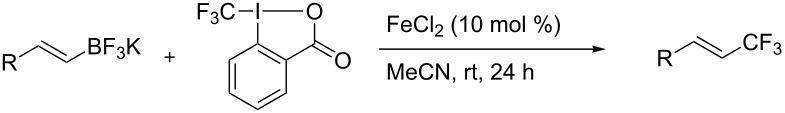					

Compound	Yield (%)	Compound	Yield (%)	Compound	Yield (%)

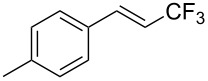	70	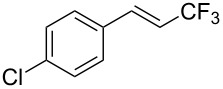	78	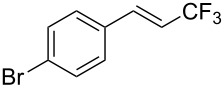	75
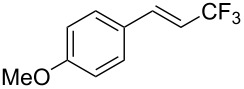	68	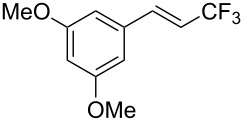	70	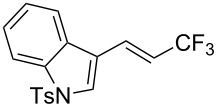	65
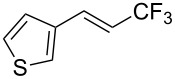	65	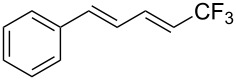	49	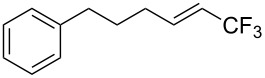	74
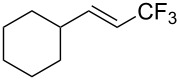	34	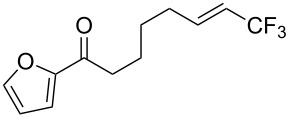	66	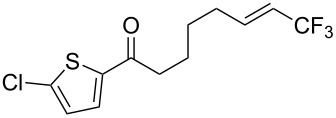	79

**3.3.6 Ag-catalyzed fluorodecarboxylation for the synthesis of trifluoromethylarenes.** An alternative approach to access trifluoromethyl arenes without the use of trifluoromethylating reagents rely on an aryl CF_2_–F bond disconnection. A clever example of this strategy has been described by V. Gouverneur et al. starting from aryl difluoroacetic acids [117]. The latters can react with Selectfluor^®^ and a catalytic amount of silver nitrate with good functional groups tolerance including ether, halide, ketone and amide. However, the presence of electron-withdrawing groups on the aromatic ring significantly decreases the yield of the transformation (Table 40). The benzylic radical generated during the reaction is probably stabilized by the two geminal fluorine atoms, by adopting an all planar geometry [118].

**Table 40:** Ag-catalyzed fluorodecarboxylation for the synthesis of trifluoromethylarenes [117].

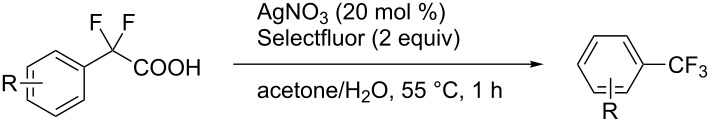					

Compound	Yield (%)	Compound	Yield (%)	Compound	Yield (%)

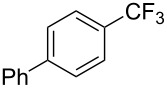	86	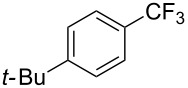	77	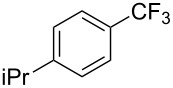	66
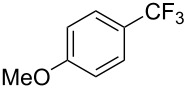	82	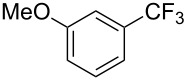	86	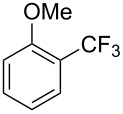	88
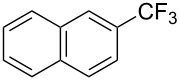	51	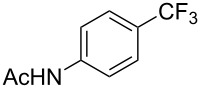	86	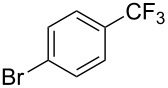	49
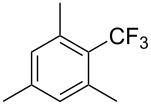	56	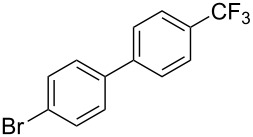	83	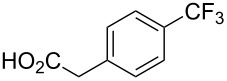	17
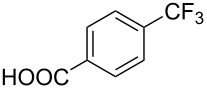	49	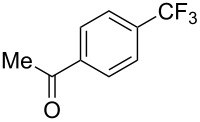	21	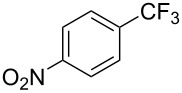	24

**Scheme 13:**
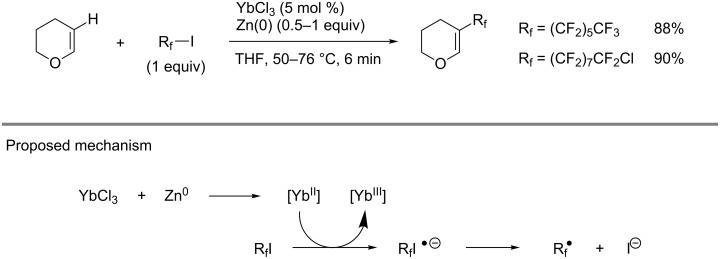
Ytterbium-catalyzed perfluoroalkylation of dihydropyran with perfluoroalkyl iodide (Y. Ding et al.) [119].

**3.3.7 Miscellaneous metals in the catalyzed perfluoroalkylation of Csp^2^–H bonds.** In 1993, Y. Ding et al. described an ytterbium-catalyzed hydroperfluoroalkylation of alkenes with perfluoroalkyl iodides. Among them, dihydropyran led instead to the product of C–H perfluoroalkylation β to the oxygen atom [119]. The reaction proceeded in the presence of Zn dust, which was believed to serve as a reductant for the in situ generation of Yb(II) species. The latter would then be able to transfer an electron to the perfluoroalkyl iodide and generate the corresponding radical (Scheme 13).

Titanium dioxide was used as heterogeneous photocatalyst in the perfluoroalkylation of α-methylstyrene with perfluorohexyl iodide by M. Yoshida et al. [120]. While the main product arose from the formal perfluoroalkylation of a methyl sp^3^-C–H bond, a byproduct corresponding to the functionalization of a methylene sp^2^-C–H bond was also obtained. The authors later applied this methodology to the perfluoroalkylation of arene C–H bonds (Table 41) [121]. The addition of methanol as an additive appeared critical playing the role of “hole shuttle”, and balancing the electron transfer to the perfluoroalkyl iodide.

**Table 41:** TiO_2_-photocatalytic perfluoroalkylations of benzenes [121].

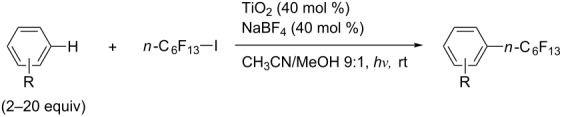			

Product	Yield (%)^a^	Product	Yield (%)^a^

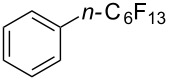	51^b^	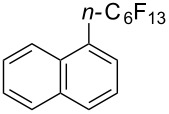	44^c^
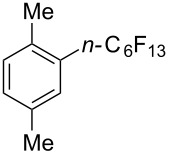	72^b^	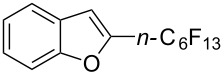	43
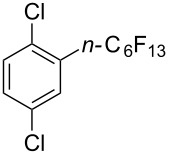	13^b^		

^a^Isolated yields based on the starting perfluorohexyl iodide, unless otherwise noted. ^b^HPLC yield. ^c^6:1 isomer mixture; the major isomer is represented.

In 2010, A. Togni and coworkers studied the trifluoromethylation of pyrroles, indoles, and various other heteroarenes or arenes in the presence of zinc salts, and with Togni’s hypervalent iodine reagents as the CF_3_-source. Yields were highly dependent on the nature of the substrate; zinc catalysts were even sometimes detrimental to the reaction, because they facilitated the competitive decomposition of the starting material [122].

**Figure 21:**
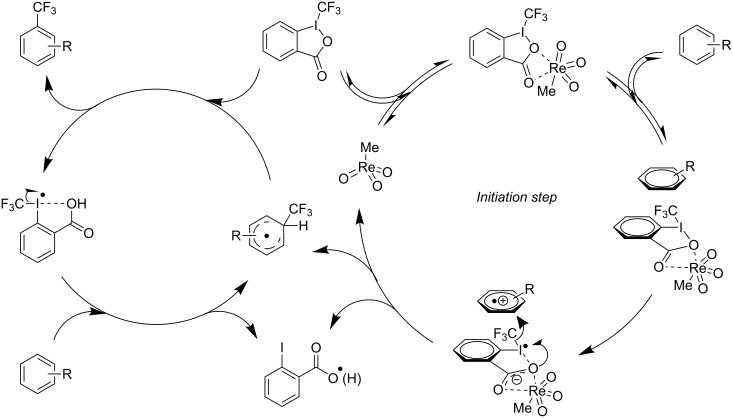
Mechanistic proposal by A. Togni et al. for the rhenium-catalyzed trifluoromethylation of arenes and heteroarenes with hypervalent iodine reagents [113].

A more successful approach was later devised by the same group [113]. With methyltrioxorhenium as a catalyst and Togni’s benziodoxolone reagent, a wide scope of aromatic and heteroaromatic compounds was trifluoromethylated with modest to good yields; even ferrocene could serve as substrate and was trifluoromethylated on one of the Cp rings. Mixtures of isomers were obtained for unsymmetrical starting materials; for instance, anisole and chloro- or iodobenzene gave an *ortho* > *para ≈ meta* preferential order of substitution, while toluene, acetophenone, *N*,*N*-dimethylaniline or nitrobenzene afforded the *para*-substituted compound as the major product. The reaction could be monitored by EPR, which showed an induction period and demonstrated the involvement of radical species in the reaction. The authors proposed a mechanism accounting for the EPR profile of the reaction and for the results of kinetic isotope effect experiments (Figure 21). In this mechanism, rhenium intervenes in the initiation step. It acts as a Lewis acid and activates the hypervalent iodine reagent, which is thus able to accept an electron by the substrate; this leads to the formation of a caged pair (aryl cation radical/reduced Togni’s reagent–rhenium complex), where iodine then transfers a CF_3_^−^ anion to the aryl cation. This recent methodology has already been applied the same year by others for the synthesis of trifluoromethylated corannulenes [123].

We discussed earlier the influence of copper sulfate on the trifluoromethylation of heteroarenes with Langlois’s reagent in the presence of *tert*-butyl peroxide (P. S. Baran et al.) [89]. In the same paper, the authors showed that cobalt perchlorate could also improve the yield of the uncatalyzed reaction. Iron sulfate, on the other hand, gave the same yield as in the absence of added metals.

### 4 Catalytic trifluoromethylthiolation

Aryl trifluoromethyl sulfides (ArSCF_3_) play an important role in pharmaceutical [124] and agrochemical research [16,125]. The trifluoromethylthio group belongs to the most lipophilic substituents as expressed by the Hansch lipophilicity parameter (π = 1.44) [126–129] and the high electronegativity of the SCF_3_ group improves significantly the stability of molecules in acidic medium. One can place this substituent next to the ever-present CF_3_ and the emerging OCF_3_ substituent [55–56,130]. In contrast, aryl trifluoromethyl sulfides are key intermediates for the preparation of trifluoromethyl sulfoxides or sulfones.

Aryl trifluoromethyl sulfides can be obtained via reaction of trifluoromethylthiolate with an electrophile like aryl halides. On the other hand, they can also be obtained by reacting aryl sulfides or disulfides under nucleophilic or radical conditions with a trifluoromethylation reagent [16,55,124]. Very recently, several elegant approaches dealing with the direct introduction of the SCF_3_-moiety have been developed in this field [131–133].

#### 4.1 Palladium catalysis

S. L. Buchwald reported on the Pd-catalyzed reaction of aryl bromides with a trifluoromethylthiolate. Good to excellent yields of aryl trifluoromethyl sulfides have been achieved under mild conditions and the reaction has been extended to a wide range of aryl- and heteroaryl bromides (Table 42) [134]. This approach employs AgSCF_3_ as SCF_3_ source in order to circumvent the fact that many convenient SCF_3_ salts are thermally unstable.

**Table 42:** Pd-catalyzed reaction of aryl bromides with trifluoromethylthiolate [134].

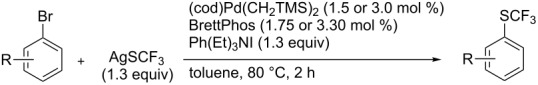					

Compound	Yield (%)	Compound	Yield (%)	Compound	Yield (%)

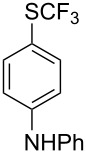	98	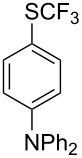	98	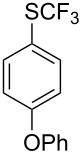	97
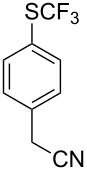	97	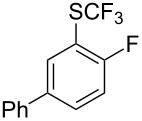	96	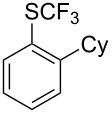	93
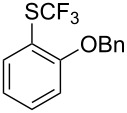	96	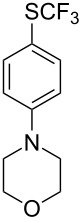	99	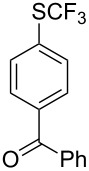	83
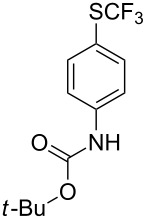	91	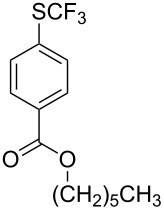	98	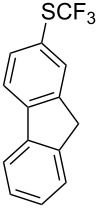	97
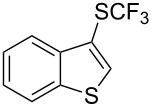	94	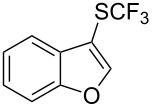	81	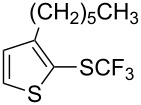	93
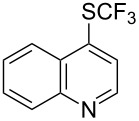	96	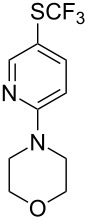	98	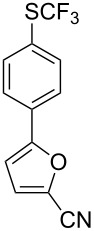	96
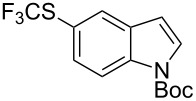	98				

The drawbacks of this approach are the use of an expensive ligand, an expensive palladium salt, a quaternary ammonium additive, and a stoichiometric amount of an expensive silver SCF_3_ derivative.

#### 4.2 Copper catalysis

F.-L. Qing was the first to report on a copper-catalyzed oxidative trifluoromethylthiolation of arylboronic acids with the Ruppert–Prakash reagent TMSCF_3_ and elemental sulfur (Table 43) [135]. This protocol is quite efficient, simple and allows for large functional group compatibility under mild reaction conditions. Another strength of the approach is that easily accessible starting materials are employed in presence of a "green" inexpensive catalyst system.

**Table 43:** Cu-catalyzed oxidative trifluoromethylthiolation of aryl boronic acids with TMSCF_3_ and elemental sulfur [135].

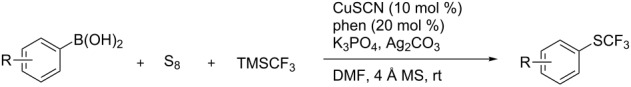					

Compound	Yield (%)	Compound	Yield (%)	Compound	Yield (%)

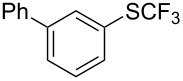	82	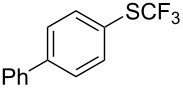	64	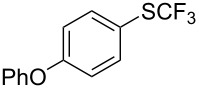	91
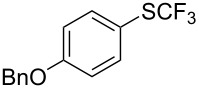	86	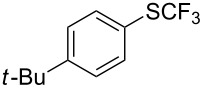	84	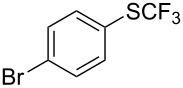	84
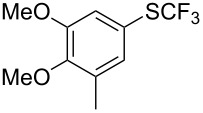	90	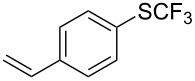	78	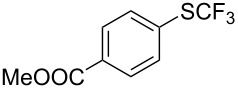	67
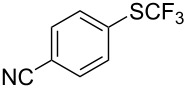	70	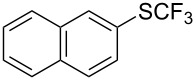	89	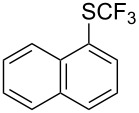	71
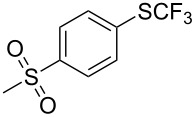	61	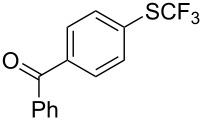	58	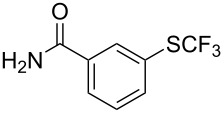	66

**Figure 22:**
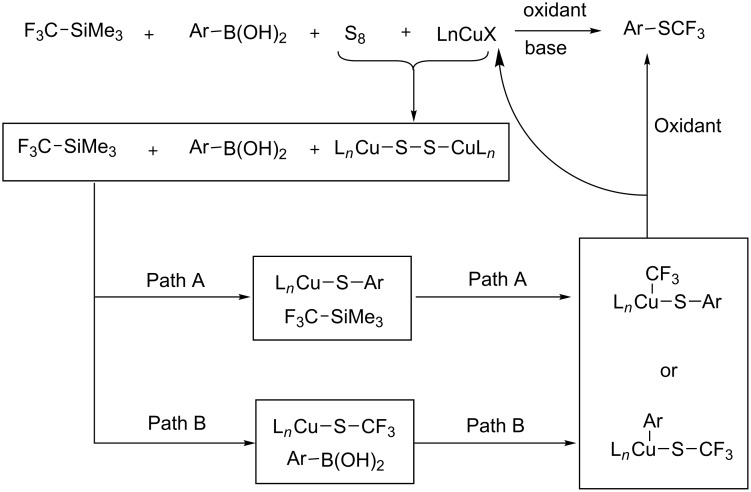
Mechanism of the Cu-catalyzed oxidative trifluoromethylthiolation of arylboronic acids with TMSCF_3_ and elemental sulfur [135].

The putative mechanism is based on the formation of a Cu(I) disulfide complex generated in situ, which reacts with arylboronic acids and TMSCF_3_ according to two possible pathways A and B (Figure 22) leading to the intermediate complex L*_n_*Cu(CF_3_)(SAr) or L*_n_*Cu(Ar)(SCF_3_), respectively. Oxidation and reductive elimination gives then the expected aryl trifluoromethyl thioether.

O. Daugulis reported on the copper-catalyzed trifluoromethylthiolation via C–H activation of 8-aminoquinoline acid amides in presence of disulfide reagents and Cu(OAc)_2_ in DMSO (Table 44) [136]. The use of inexpensive copper acetate and the removable directing group are significant advantages of this approach. Bromide, ester, and chloride functionalities are tolerated and the reaction has been applied to aromatic as well as five- and six-membered heterocyclic substrates.

**Table 44:** Cu-catalyzed trifluoromethylthiolation via C–H activation [136].

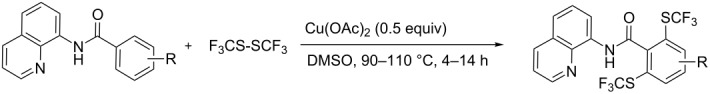			

Compound	Yield (%)	Compound	Yield (%)

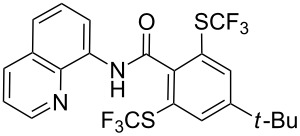	76	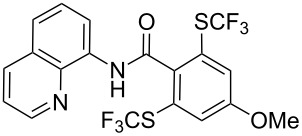	67
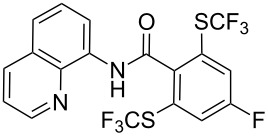	73	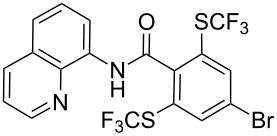	70
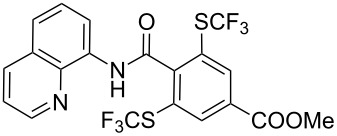	72	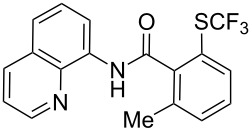	63
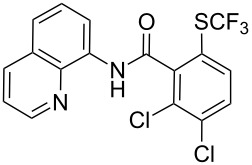	59	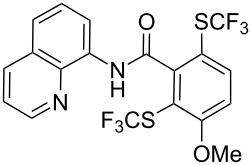	70
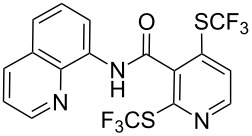	43	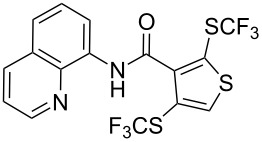	59

**Scheme 14:**
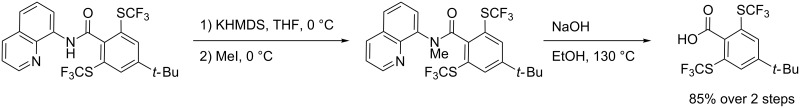
Removal of the 8-aminoquinoline auxiliary [136].

The 8-aminoquinoline auxiliary can be easily removed affording the trifluoromethylthiolated acid (Scheme 14).

L. Lu and Q. Shen reported on the use of an electrophilic trifluoromethylthio reagent based on Togni's hypervalent iodine reagent for trifluoromethylation reactions (Table 45) [137]. Trifluoromethylthiolation of various substrates, such as β-ketoesters, aldehydes, amides, aryl, or vinyl boronic acids, or alkynes, have been achieved under mild conditions.

**Table 45:** Cu-catalyzed trifluoromethylthiolation of boronic acids employing a hypervalent iodine reagent [137].

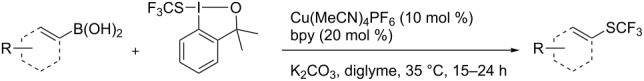					

Compound	Yield (%)	Compound	Yield (%)	Compound	Yield (%)

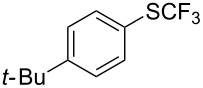	90	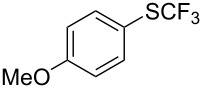	92	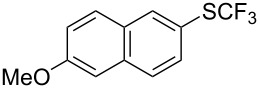	95
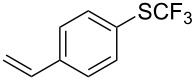	89	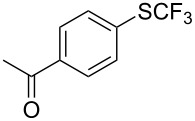	87	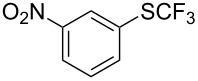	64
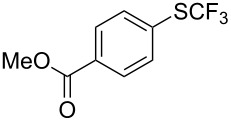	58	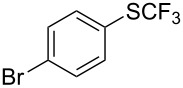	87	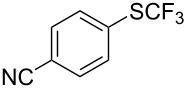	58
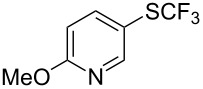	65	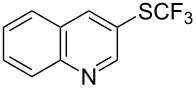	40	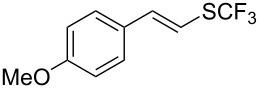	75
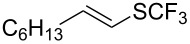	57				

In order to avoid the preparation of trifluoromethylthiolation reagents by trifluoromethylations of sulfides, N. Shibata studied an approach based on the use of the easily accessible trifluoromethanesulfonyl (CF_3_SO_2_) unit which is stable and often found in commonly used organic reagents such as CF_3_SO_2_Cl, CF_3_SO_2_Na, CF_3_SO_3_H, and (CF_3_SO_2_)_2_O. They designed a new electrophilic-type trifluoromethylthiolation reagent, a trifluoromethanesulfonyl hypervalent iodonium ylide [138]. It is easily synthesized in quantitative yield by the reaction of α-trifluoromethanesulfonyl phenyl ketone and phenyliodine(III) diacetate (PIDA).

In the presence of a catalytic amount of copper(I) chloride, this reagent trifluoromethyltiolates a wide variety of nucleophiles like enamines, β-keto esters and indoles allowing the C-sp^2^ trifluoromethylthiolation of vinylic C–H (Table 46) and aromatic (Table 47) bonds.

**Table 46:** Cu-catalyzed trifluoromethylthiolation of vinylic C–H bonds with a trifluoromethanesulfonyl hypervalent iodonium ylide [138].

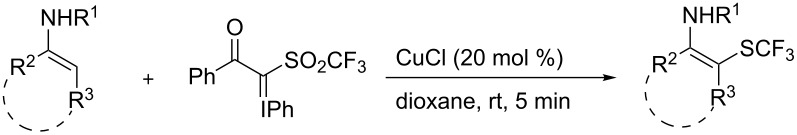			

Compound	Yield (%)	Compound	Yield (%)

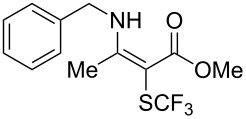	92	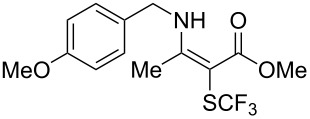	89
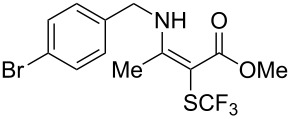	82	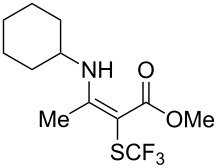	89
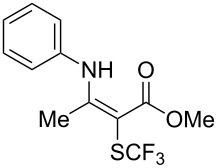	77	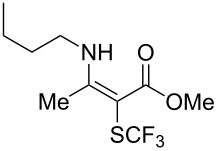	75
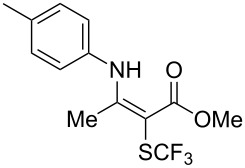	88	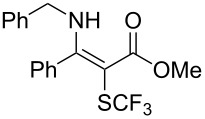	90
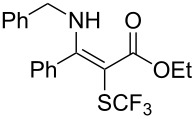	87	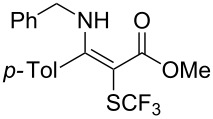	94
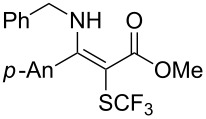	96	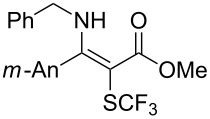	94
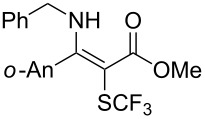	94	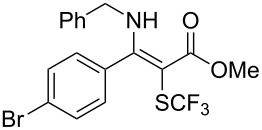	84
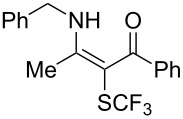	97	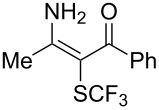	84
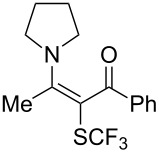	74	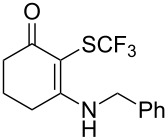	84

**Table 47:** Cu-catalyzed trifluoromethylthiolation of aromatic C–H bonds with a trifluoromethanesulfonyl hypervalent iodonium ylide [138].

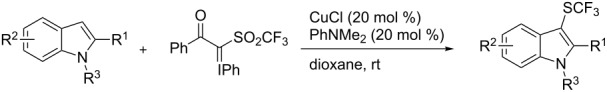					

Compound	Yield (%)	Compound	Yield (%)	Compound	Yield (%)

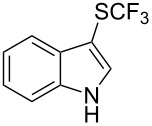	83	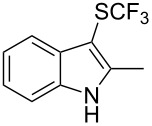	83	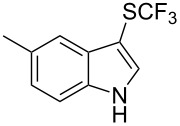	6%
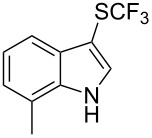	73	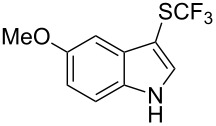	36	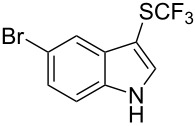	71
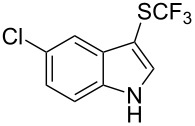	52	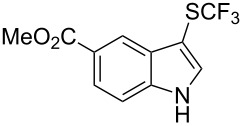	32	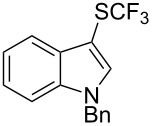	84

**Figure 23:**
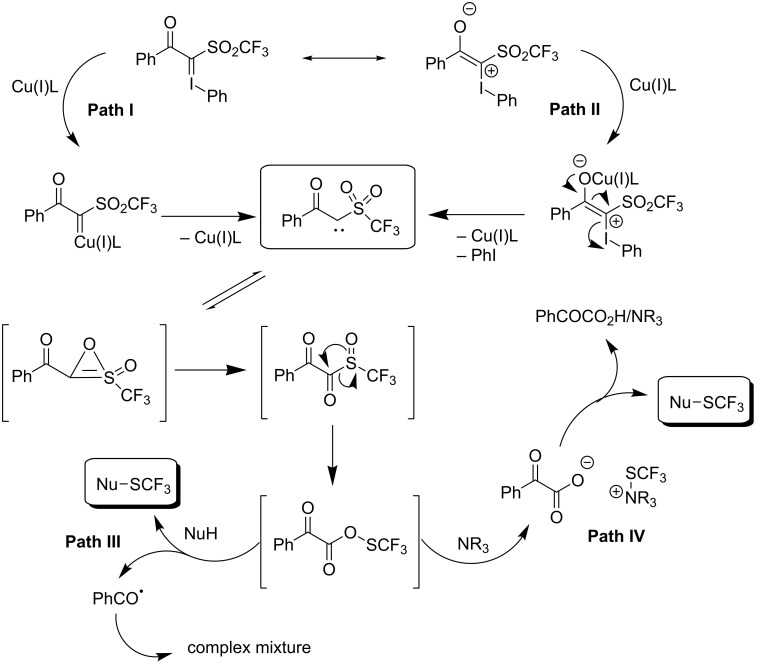
Mechanism of the Cu-catalyzed trifluoromethylthiolation of C–H bonds with a trifluoromethanesulfonyl hypervalent iodonium ylide [138].

The reasonable mechanism for this reaction is shown in Figure 23. A copper carbenoid may initially be formed and decompose to a sulfonyl carbene (Path I, Figure 23). Or, the reagent could be activated by a copper(I) salt and generate a zwitterionic intermediate, which eliminates iodobenzene to form a carbene (Path II). Next, an oxirene (in equilibrium with carbene) rearranges to sulfoxide and collapses to the true reactive species, thioperoxoate. Electrophilic transfer trifluoromethylthiolation to the nucleophile then yields the desired products (Path III). In presence of an amine, a trifluoromethylthiolated ammonium salt might be formed which is subsequently attacked by the nucleophile yielding the final product (Path IV).

#### 4.3 Nickel catalysis

D. A. Vicic studied the use of the cheaper and more soluble [NMe_4_][SCF_3_] reagent instead of AgSCF_3_ used by S. L. Buchwald in his studies [125]. However, one major constraint in the use of this reagent is that transition metal-catalyzed reactions have to be realized under extremely mild and anhydrous conditions. This inspired this group to employ a bipyridine nickel system as a catalyst in order to activate aryl halides at room temperature. They could show that the nickel catalyst allows the efficient incorporation of the SCF_3_ functionality into a variety of aryl halides. Electron-rich aryl halides were better substrates than electron-poor analogues (Table 48).

**Table 48:** Ni-catalyzed trifluoromethylthiolation of aryl halides with [NMe_4_][SCF_3_] [125].

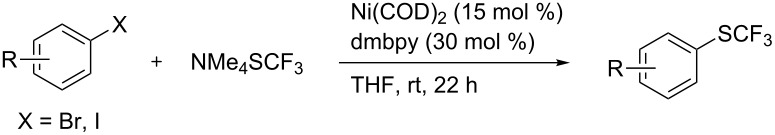					

Compound	Yield (%)	Compound	Yield (%)	Compound	Yield (%)

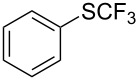	Cl: 0 Br: 65	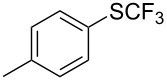	I: 90	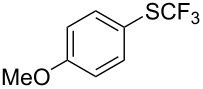	I: 90
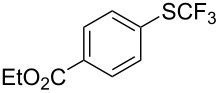	I: 45	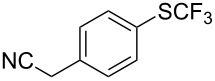	I: 47	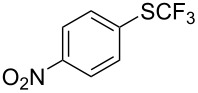	I: 0
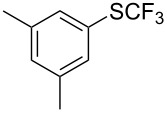	I: 83	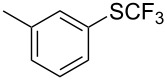	Br: 37	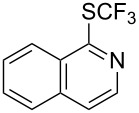	I: 55
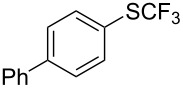	Br: 64 I: 92	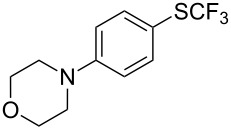	I: 91		

## Conclusion

Over the last two years or so, organofluorine chemistry has made an important step forward by adding transition metal catalysis to its toolbox, to the benefit of chemists working in pharmaceuticals, agrochemicals and material sciences or diagnosis. Reactions that have been unimaginable some years ago have been the focus of researchers, many of them not necessarily experts in fluorine chemistry. In particular the organometallic chemistry community has contributed significantly. Despite this exciting progress, the catalytic introduction of fluorine and fluorinated groups is still in its infancy and much skill needs to be revealed regarding mechanism, the nature and amount of the metal employed and scale up of reactions for industrial applications.

This "Small atom with a big ego" (title of the ACS Symposium in San Francisco in 2000) will without any doubt continue to have a brilliant future.
